# Supplementary material for: GM-CSF Calibrates Macrophage Defense and Wound Healing Programs during Intestinal Infection and Inflammation
Source: Cell Rep. 2020 Jul 7;32(1):107857. doi: 10.1016/j.celrep.2020.107857 (PMC7351110; doi:10.1016/j.celrep.2020.107857)
Supplement: Document S1. Figures S1–S6 [file mmc1.pdf]

Cell Reports, Volume *32*

Supplemental Information

GM-CSF Calibrates Macrophage Defense
and Wound Healing Programs
during Intestinal Infection and Inflammation

Tomas Castro-Dopico, Aaron Fleming, Thomas W. Dennison, John R. Ferdinand, Katherine Harcourt, Benjamin J. Stewart, Zaeem Cader, Zewen K. Tuong, Chenzhi Jing, Laurence S.C. Lok, Rebeccah J. Mathews, Anaïs Portet, Arthur Kaser, Simon Clare, and Menna R. Clatworthy

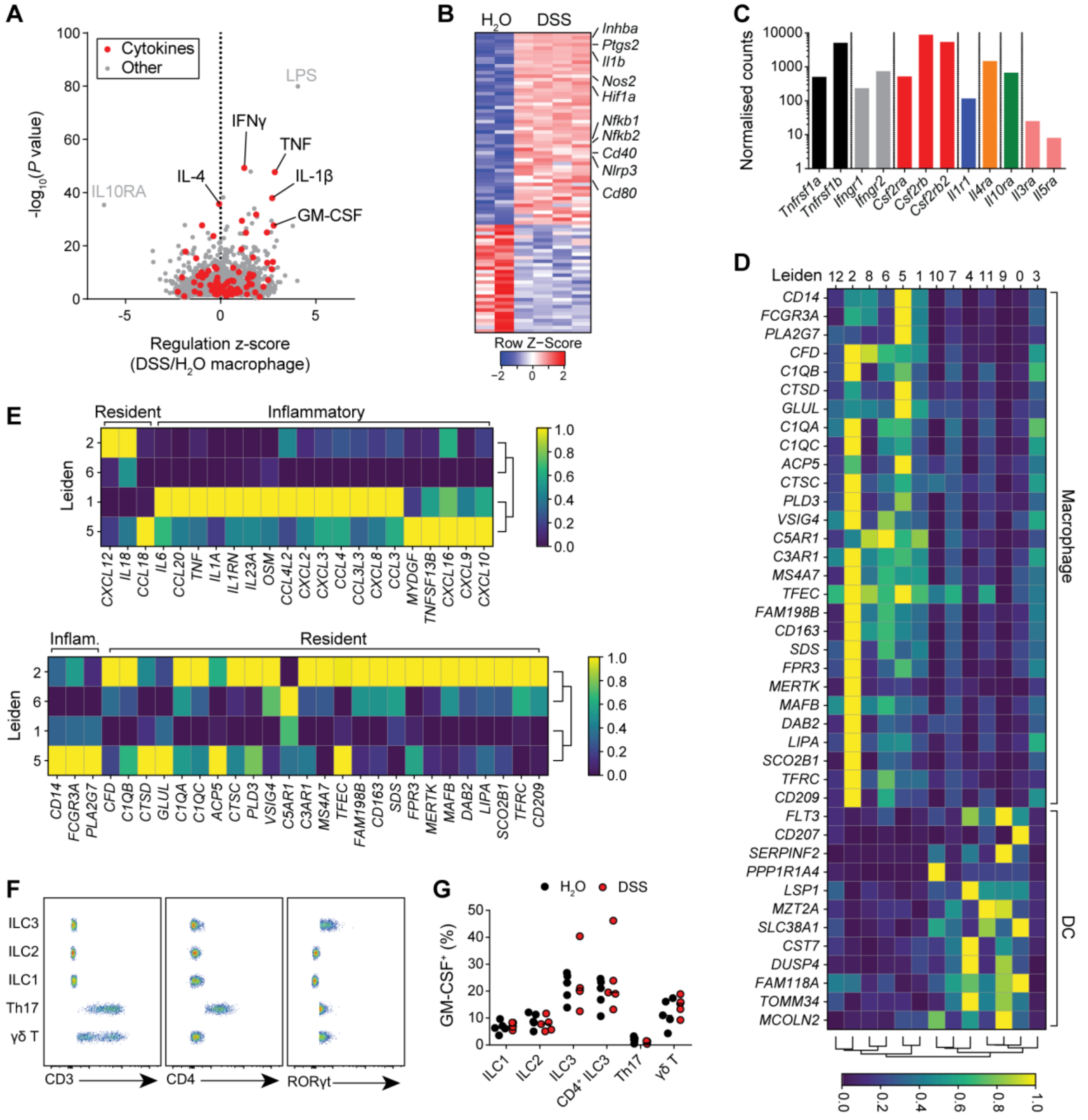

Figure S1. GM-CSF is an upstream regulator of inflammatory macrophage function in the intestine. Related to Figure 1. (**A**) Plot of z-score and *P* value for predicted regulators of DSS vs H_2O macrophage activation, determined by IPA. Data derived from GEO: GSE109040. (**B**) Heatmap of predicted GM-CSF regulated genes in DSS macrophages from dataset in **A**. (**C**) Normalised expression counts of candidate cytokine receptor genes in colonic macrophages from **A**. (**D, E**) Heatmaps of marker genes and cytokines distinguishing (**D**) macrophages from dendritic cells and (**E**) resident from inflammatory macrophages amongst all MNPs in human mucosal biopsies from patients with ileal Crohn's disease, determined by scRNA-seq. Data derived from GEO: GSE134809. (**F**) Surface marker and RORγt expression by colonic ILC and T cell subsets. (**G**) GM-CSF$^+$ cells as a proportion of each immune cell subset from mice treated with H_2O or 2 % DSS for 6 days. N = 5 per group. Medians indicated. Data (**F, G**) representative of two independent experiments. *P* values were calculated in IPA software (**A**).

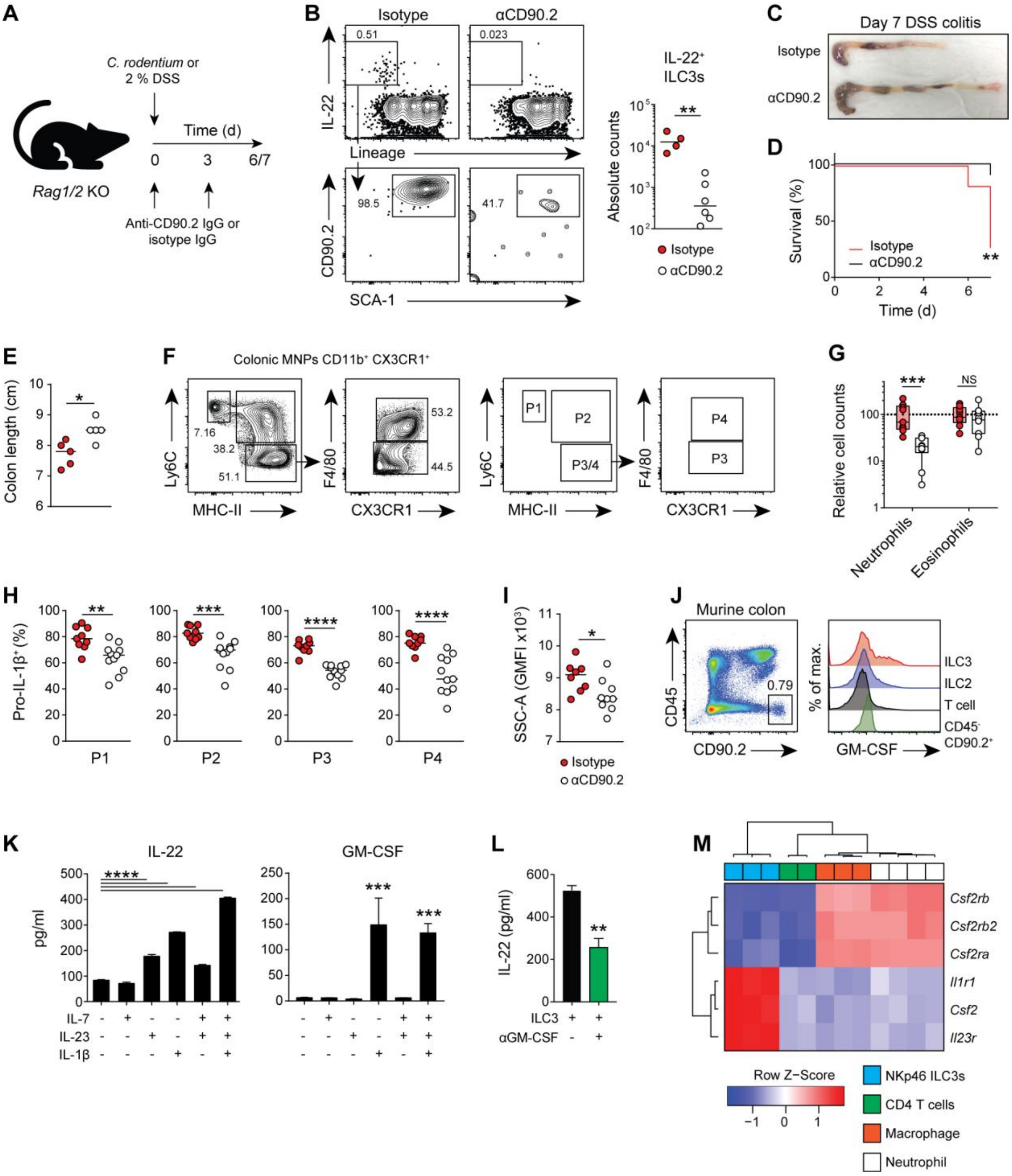

Figure S2. ILCs and innate GM-CSF augment intestinal macrophage activation during enteropathic infection and colitis. Related to Figure 2. (**A**) Schematic of ILC depletion strategy. (**B**) Colonic lineage^{-} CD90.2^{+} SCA-1^{+} IL-22^{+} ILC3 quantification in 2 % DSS-treated *Rag2*$^{-/-}$ mice, as in **A** (*n* = 4-6 per group). Medians indicated. (**C**) Colon length in *Rag2*$^{-/-}$ mice treated as in **A** at day 7 after 2 % DSS administration. (**D**) Survival of mice treated as in **C** (exceeding 20 % weight loss). *N* = 10-11 per group. (**E**) Colon length in mice treated as in **A**, infected with *C. rodentium* at 7 dpi. *N* = 5 per group. Medians indicated. (**F**) Representative MNP waterfall gating strategy in murine colon. (**G**) Colonic neutrophil and eosinophil relative cell count in *Rag2*$^{-/-}$ mice treated with 2 % DSS ± isotype or anti-CD90.2 IgG for 6 days. *N* = 9-11 per group. Medians indicated. (**H**) Pro-IL-1β expression by colonic MNP subsets (P1-P4) in mice treated as in **B**. Medians indicated. (**I**) SSC of colonic Ly6C^{lo} MHC-II^{+} MNPs from mice treated as in **A** with *C. rodentium*. Medians indicated. (**J**) GM-CSF expression by non-haematopoietic CD90.2^{+} CD45.2^{-} cells in murine colon. Mice treated as in **A**. (**K**) IL-22 and GM-CSF production by flow-sorted intestinal ILC3s cultured with indicated cytokines for 16h. Mean ± SEM indicated. (**L**) IL-22 production in ILC3-BMDM co-cultures ± isotype or anti-GM-CSF IgG after 16h. Mean ± SEM indicated. (**M**) Cytokine and receptor expression by intestinal NKp46^{+} ILC3s, lymph node CD4^{+}

T cells, lung macrophages and blood neutrophils from the Immgen Consortium Phase 2 (GEO: GSE37448). Data are representative of three independent experiments (**B-I, K, L**), with single (**B, C, E-G, I, K, L**) or two pooled experiments (**D, G, H**) shown. *P* values were calculated using Mann-Whitney U test (**B, E, G-I**), Gehan-Breslow-Wilcoxon test (**D**), a one-way ANOVA with Tukey's multiple comparisons test (**K**), and Student's *t* test (**L**). * $P < 0.05$; ** $P < 0.01$; *** $P < 0.001$; **** $P < 0.0001$.

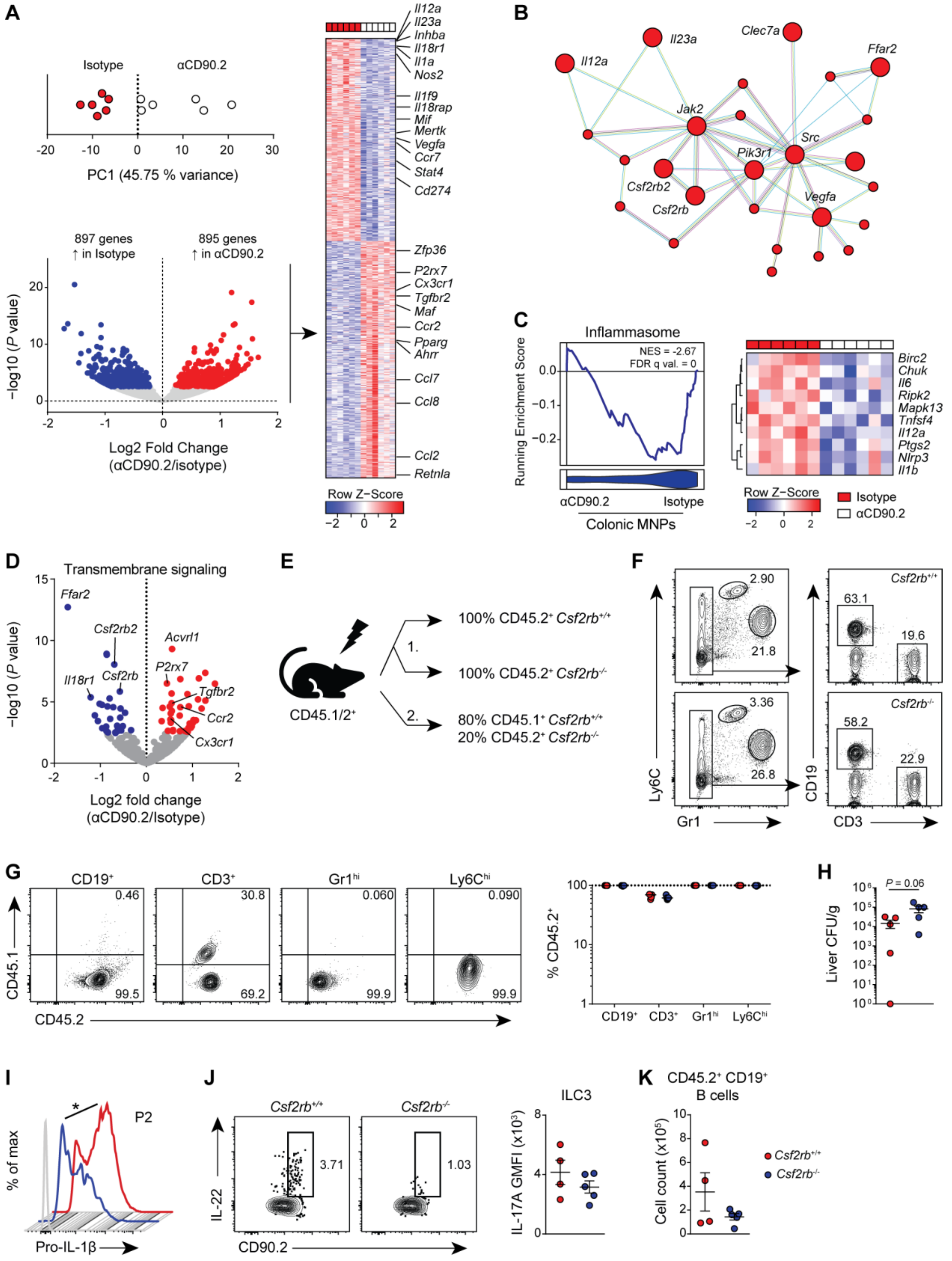

Figure S3. GM-CSF drives inflammatory macrophage function and type 17 immunity *in vivo*. Related to Figure 3. (**A**) Principal component 1 of RNAseq dataset of colonic Ly6C^{lo} MHC-II^{+} macrophages from *Rag2*$^{-/-}$ mice treated with 2 % DSS ± isotype or anti-CD90.2 IgG antibodies (top), and volcano plot and heatmap of all DEGs (adj. *P* val. < 0.05). Selected genes of interest are highlighted. *N* = 6 per group. (**B**) STRING analysis of gene networks (highest confidence = 0.900; no interactors) among top 200 down-regulated differentially expressed genes (DEGs = adj. *P* value < 0.05) from colonic macrophages in **A**. (**C**) RNAseq gene set enrichment analysis (GSEA) of inflammasome genes in colonic Ly6C^{lo} MHC-II^{+} macrophages in mice treated

as in **c**. $N = 6$ per group. Top 10 leading edge genes shown in heatmap. (**D**) Volcano plot of transmembrane signalling genes (GO:0004888) in colonic macrophages from **A**. (**E**) Bone marrow chimera generation strategy. (**F, G**) Blood reconstitution analysis in 100 % bone marrow chimeric mice at 8 weeks post-irradiation. $N = 5$ per group. Medians indicated. (**H**) Liver CFU in 100 % bone marrow chimeras at 10 dpi. $N = 5$ per group. Mean ± SEM indicated. (**I**) Colonic P2 MNP pro-IL-1β expression in 100 % chimeric mice shown in **F**. Mean ± SEM indicated. (**J**) IL-22^{+} gating and IL-17A quantification of donor colonic CD3ϵ^{-} CD4^{-} ILCs in 100 % chimeric mice at 10 dpi. Mean ± SEM indicated. (**K**) Colonic donor CD19^{+} B cell quantification from mice in **J**. Data representative of two independent experiments. P values were calculated using the standard DESeq 2 method with multiple comparisons correction using the BH procedure (**A, D**), Student's t test (**H**), and nonparametric Mann-Whitney U test (**I**). * $P < 0.05$; ** $P < 0.01$; *** $P < 0.001$; **** $P < 0.0001$.

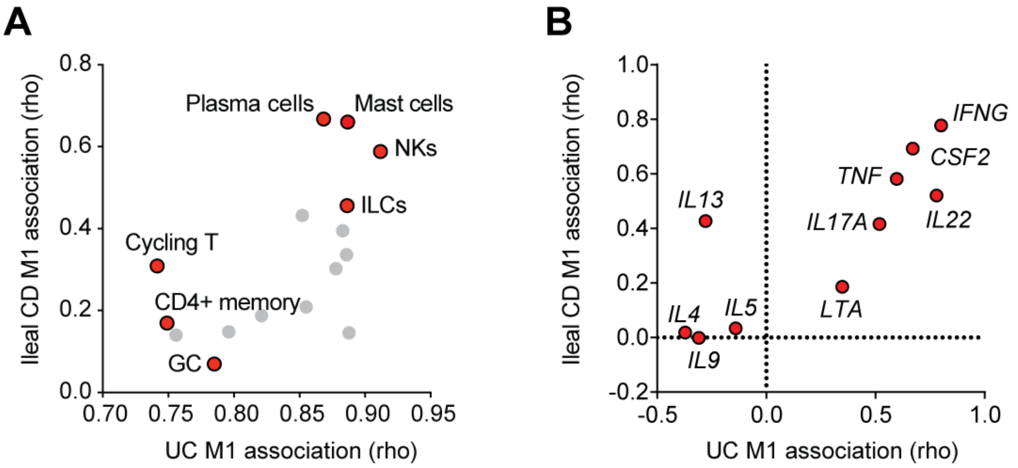

Figure S4. ILCs and GM-CSF regulate intestinal macrophage polarisation. Related to Figure 4. (**A, B**) Immune cell signature (**A**) and cytokine expression (**B**) rho values for correlation with sLPS M1 signature in transcriptomics datasets of UC (GEO: GSE59071) and ileal CD (GEO: GSE93624) mucosal biopsies.

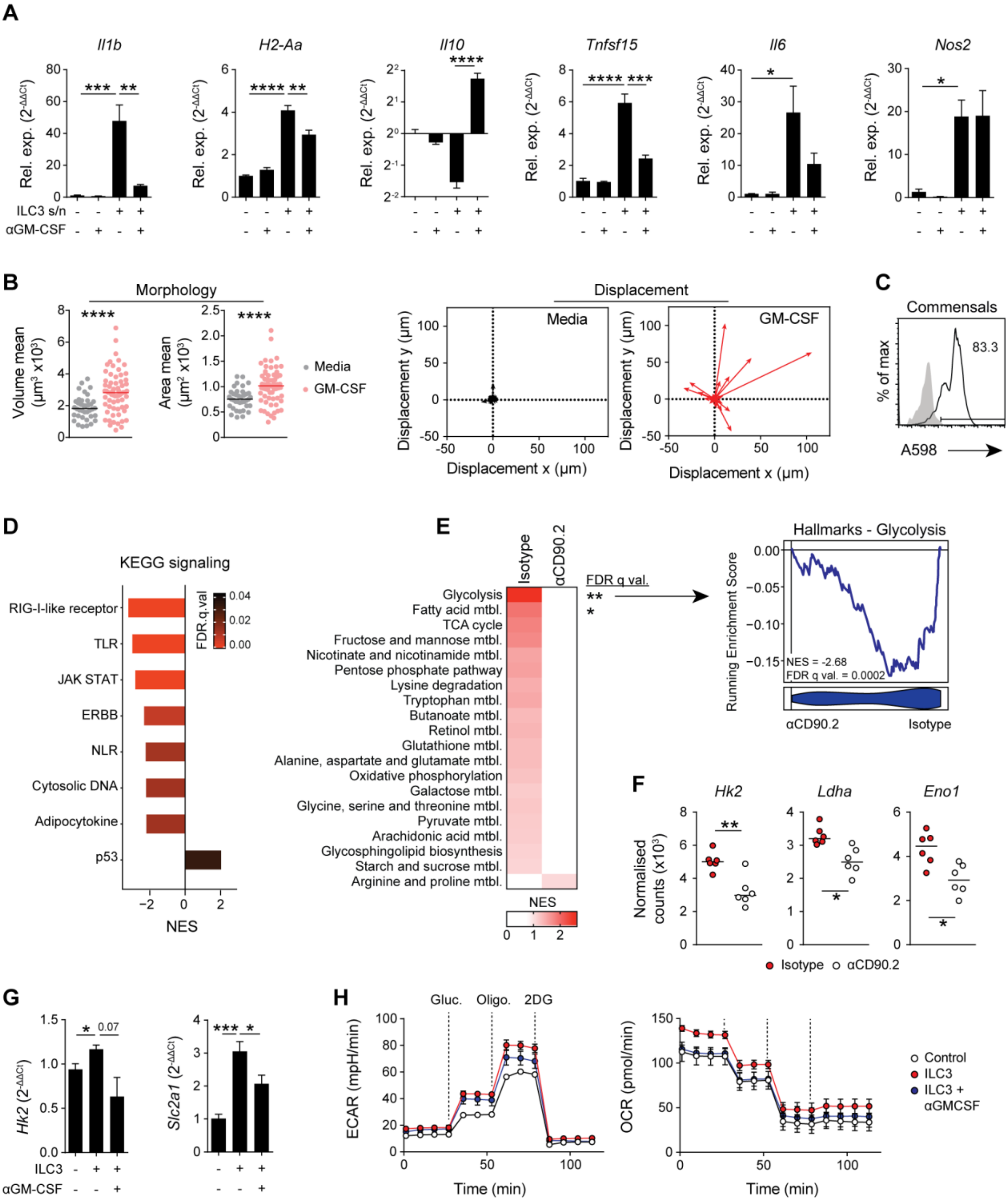

Figure S5. ILC3-derived GM-CSF regulates MNP microbicidal activity and metabolism. Related to Figure 5. (**A**) Cytokine, MHC-II, and NOS2 expression in BMDMs treated with ILC3-conditioned media ± anti-GM-CSF IgG for 16 h. Mean ± SEM indicated. (**B**) Morphology and displacement analysis of control or GM-CSF-primed BMDMs. Medians are indicated. (**C**) Fluorescent labelling of murine intestinal commensals. (**D**) KEGG signalling pathways in colonic macrophages ± anti-CD90.2 IgG administration after 6 days of DSS by RNAseq. (**E**) RNAseq GSEA of Kyoto Encyclopaedia of Genes and Genomes (KEGG) metabolic pathways in colonic Ly6C^{lo} MHC-II^{+} macrophages from *Rag2*$^{-/-}$ mice treated with 2 % DSS ± isotype or anti-CD90.2 IgG for 6 days (n = 6 per group). Medians indicated. (**F**) Expression of key glycolysis genes from macrophages in **E**. Medians indicated. (**G**) HK2 and GLUT-1 expression in BMDMs ± ILC3-conditioned supernatant for 16h ± anti-GM-CSF IgG. Mean ± SEM indicated. (**H**) ECAR and OCR trace in BMDMs ± ILC3-conditioned supernatant ± anti-GM-CSF IgG for 16 h. Mean ± SEM indicated. Data are representative of two (**B, H**) or three (**A, G**) independent experiments. P values calculated using a one-way ANOVA (**A, G**), Mann-Whitney U test (**B**). * P < 0.05; ** P < 0.01; *** P < 0.001; **** P < 0.0001.

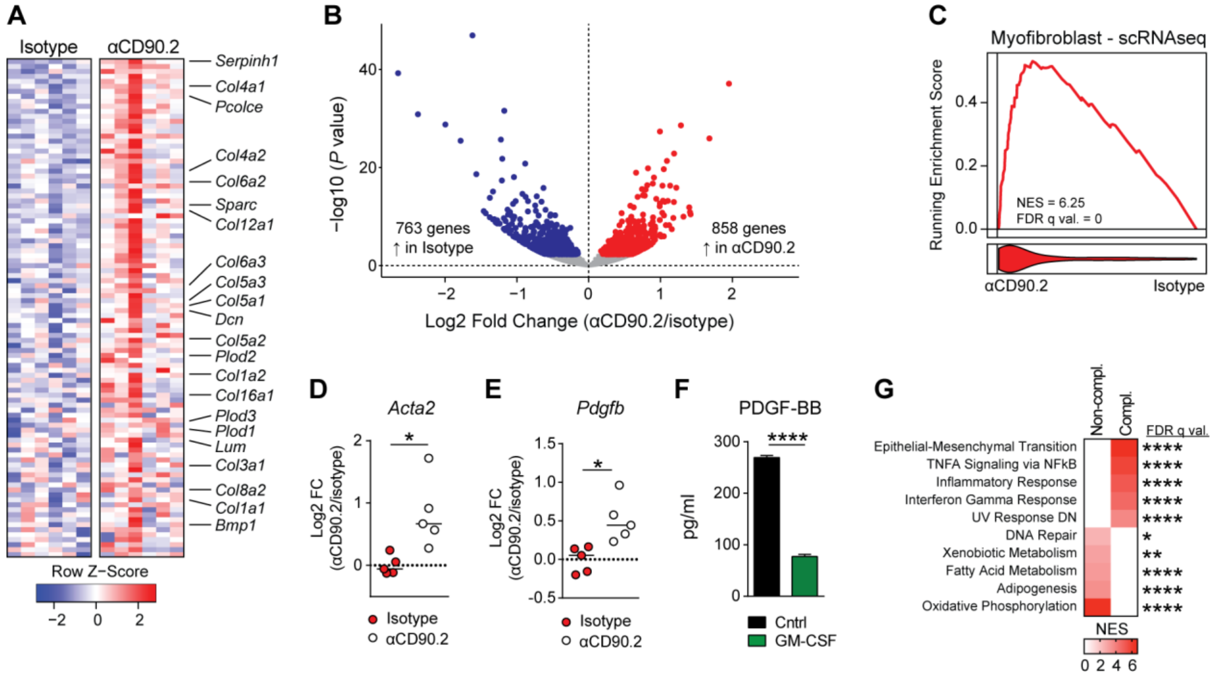

Figure S6. ILCs suppress a pro-repair macrophage phenotype. Related to Figure 6. (**A**) Heatmap of *Epithelial-Mesenchymal Transition* leading edge genes showing enrichment in colonic Ly6C^{lo} MHC-II^{+} macrophages from *Rag2*$^{-/-}$ mice treated with 2 % DSS ± anti-CD90.2 IgG for 6 days (n = 6 per group). Annotated genes are involved in collagen formation or interaction. (**B**) Volcano plot of DEGs in whole colonic tissue from mice treated as in **A**. (**C**) Myofibroblast gene signature GSEA in whole colonic tissue from mice treated as in **A**. (**D**) *Acta2* expression in whole colonic tissue from mice in **A**. Medians are indicated. (**E**) *Pdgfb* expression in whole colon tissue determined by RNAseq in mice treated as in **A**. Medians indicated. (**F**) PDGF-BB ELISA on supernatants from BMDMs treated with control or GM-CSF-supplemented media for 24 h. Mean ± SEM indicated. (**G**) GSEA of Hallmarks pathways in a publicly available transcriptomics dataset of complicated (n = 27) versus non-complicated (n = 183) ileal Crohn's disease biopsies, derived from GEO: GSE93624. Data representative of three independent experiments (**F**). P values were calculated by DESeq 2 method with multiple comparisons correction using the BH procedure (**A, B, D, E**), and Student's t test (**F**).* $P < 0.05$; ** $P < 0.01$; *** $P < 0.001$; **** $P < 0.0001$.
