# Supplementary material for: GM-CSF Calibrates Macrophage Defense and Wound Healing Programs during Intestinal Infection and Inflammation
Source: Cell Rep. 2020 Jul 7;32(1):107857. doi: 10.1016/j.celrep.2020.107857 (PMC7351110; doi:10.1016/j.celrep.2020.107857)
Supplement: Document S2. Article plus Supplemental Information [file mmc4.pdf]

Cell Reports

Article

GM-CSF Calibrates Macrophage Defense and Wound Healing Programs during Intestinal Infection and Inflammation

Graphical Abstract

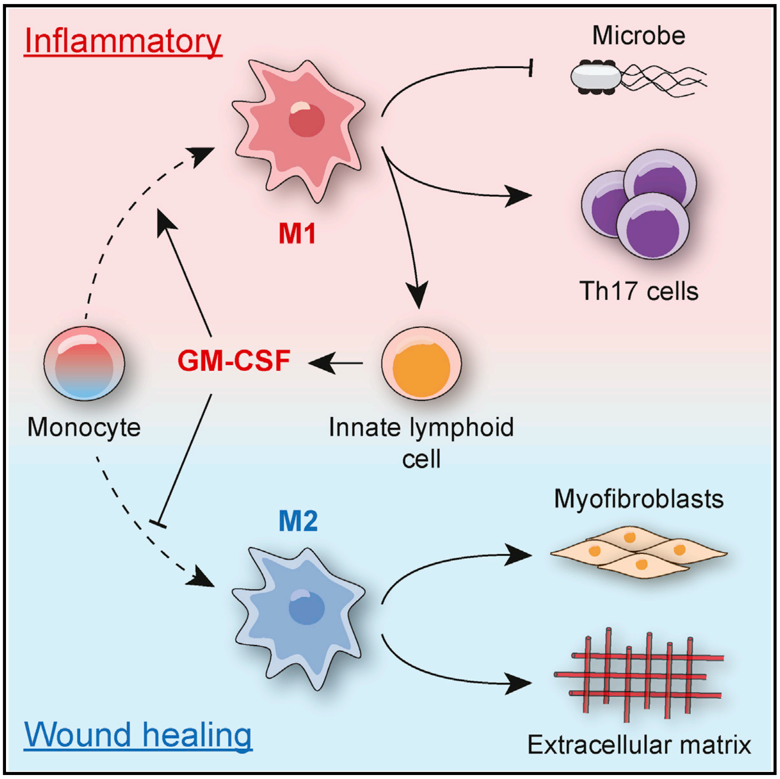

Highlights

- ILCs are a major source of GM-CSF, a critical regulator of gut inflammatory macrophages
- GM-CSF promotes ILC-macrophage crosstalk and augments type 17 and barrier immunity
- GM-CSF-induced macrophages are protective during infection but drive inflammatory colitis
- GM-CSF suppresses wound-healing macrophage response associated with intestinal fibrosis

Authors

Tomas Castro-Dopico, Aaron Fleming, Thomas W. Dennison, ..., Arthur Kaser, Simon Clare, Menna R. Clatworthy

Correspondence

mrc38@cam.ac.uk

In Brief

Castro-Dopico et al. identify GM-CSF as a major upstream regulator of intestinal inflammatory macrophages, with ILC-derived GM-CSF polarizing macrophages toward a glycolytic, anti-microbial phenotype while suppressing wound-healing, pro-fibrotic activity. GM-CSF initiates further ILC-macrophage crosstalk, amplifying ILC3 activation and type 17 immunity.

Castro-Dopico et al., 2020, Cell Reports *32*, 107857
July 7, 2020 © 2020 The Authors.
https://doi.org/10.1016/j.celrep.2020.107857

CellPress

Cell Reports

CellPress
OPEN ACCESS

Article

GM-CSF Calibrates Macrophage Defense and Wound Healing Programs during Intestinal Infection and Inflammation

Tomas Castro-Dopico,[1] Aaron Fleming,[1] Thomas W. Dennison,[1] John R. Ferdinand,[1] Katherine Harcourt,[2] Benjamin J. Stewart,[1,2] Zaeem Cader,[3] Zewen K. Tuong,[1,2] Chenzhi Jing,[1] Laurence S.C. Lok,[1] Rebeccah J. Mathews,[1] Anaïs Portet,[1] Arthur Kaser,[3] Simon Clare,[2] and Menna R. Clatworthy[1,2,4,5,*]

[1]Molecular Immunity Unit, University of Cambridge Department of Medicine, MRC Laboratory of Molecular Biology, Cambridge, UK

[2]Wellcome Sanger Institute, Hinxton, UK

[3]Division of Gastroenterology, Department of Medicine, University of Cambridge, Cambridge, UK

[4]NIHR Cambridge Biomedical Research Centre, Cambridge, UK

[5]Lead Contact

*Correspondence: mrc38@cam.ac.uk

https://doi.org/10.1016/j.celrep.2020.107857

SUMMARY

Macrophages play a central role in intestinal immunity, but inappropriate macrophage activation is associated with inflammatory bowel disease (IBD). Here, we identify granulocyte-macrophage colony stimulating factor (GM-CSF) as a critical regulator of intestinal macrophage activation in patients with IBD and mice with dextran sodium sulfate (DSS)-induced colitis. We find that GM-CSF drives the maturation and polarization of inflammatory intestinal macrophages, promoting anti-microbial functions while suppressing wound-healing transcriptional programs. Group 3 innate lymphoid cells (ILC3s) are a major source of GM-CSF in intestinal inflammation, with a strong positive correlation observed between ILC or *CSF2* transcripts and M1 macrophage signatures in IBD mucosal biopsies. Furthermore, GM-CSF-dependent macrophage polarization results in a positive feedback loop that augmented ILC3 activation and type 17 immunity. Together, our data reveal an important role for GM-CSF-mediated ILC-macrophage crosstalk in calibrating intestinal macrophage phenotype to enhance anti-bacterial responses, while inhibiting pro-repair functions associated with fibrosis and stricturing, with important clinical implications.

INTRODUCTION

The gastrointestinal tract is colonized by trillions of commensals and represents a substantial interface between the host immune system and the environment. The maintenance of this barrier requires immunological tolerance to beneficial microflora, while retaining the capacity to mount an inflammatory response to enteropathic organisms. Tissue-resident immune cells play a critical role in this process, carrying out diverse functions that are coordinated by complex inter-cellular signaling networks. Disruption of normal mucosal immune interactions is characteristic of inflammatory bowel disease (IBD), a chronic, relapsing condition that is increasing in frequency (Kaser et al., 2010). Intestinal macrophages form a central hub in this cellular network, with the ability to detect microbial and environmental stimuli and to produce cytokines and chemokines that drive local immune cells toward tolerance or inflammation (Cummings et al., 2016; Joeris et al., 2017).

Intestinal macrophages are largely replenished from circulating monocytes (Bain et al., 2014), which during homeostasis, undergo tolerogenic programming upon entry into the intestine toward an interleukin (IL)-10-producing phenotype (Bernshtein et al., 2019; Ip et al., 2017; Zigmond et al., 2014). In contrast, during intestinal inflammation, infiltrating monocytes differentiate toward an inflammatory phenotype, characterized by an altered cytokine and chemokine profile and enhanced microbicidal activity (Bain et al., 2013; Zigmond et al., 2012). Although these inflammatory macrophages are important for defense against enteropathogens (Seo et al., 2015b), inappropriate macrophage activation is associated with tissue pathology in IBD and in murine models of colitis (Castro-Dopico et al., 2019; Seo et al., 2015a). However, the factors that promote inflammatory macrophage responses in the gut remain incompletely understood.

Recent studies have highlighted the importance of macrophage-derived cytokines such as IL-1β, IL-23, and TL1A in driving type 17 immune responses, including T helper 17 (Th17) cells and group 3 innate lymphoid cells (ILC3) (Coccia et al., 2012; Longman et al., 2014; Manta et al., 2013). In response to these cytokines, ILC3s in turn produce IL-22, promoting epithelial integrity and anti-microbial peptide (AMP) production to limit system bacterial spread following challenge with enteropathic bacteria (Cella et al., 2009; Satoh-Takayama et al., 2008; Sonnenberg et al., 2011). ILC3s also produce granulocyte-macrophage colony stimulating factor (GM-CSF), stimulating

This is an open access article under the CC BY license (http://creativecommons.org/licenses/by/4.0/).

tolerogenic dendritic cells (DC) that prime regulatory T cells to maintain intestinal homeostasis (Mortha et al., 2014). During intestinal inflammation, ILC3-derived GM-CSF can be pro-inflammatory, mobilizing and activating eosinophils (Griseri et al., 2012, 2015), enhancing neutrophil and monocyte recruitment (Pearson et al., 2016; Song et al., 2015), and controlling ILC3 migration (Pearson et al., 2016).

We have previously demonstrated that anti-microbial IgG is elevated during chronic intestinal inflammation and drives detrimental macrophage responses in the context of ulcerative colitis by cross-linking macrophage Fcγ receptors (Castro-Dopico et al., 2019). In order to discover additional factors that might promote inflammatory macrophage activation during intestinal inflammation, we investigated the transcriptome of intestinal macrophages in Crohn's disease patients and in a murine model of colitis. This identified GM-CSF as a critical regulator of macrophage activation in intestinal inflammation, which was primarily produced by innate lymphocytes, particularly ILC3s. In mouse models of intestinal infection and inflammation, we found that GM-CSF regulated the maturation and polarization of intestinal macrophages, preferentially inducing anti-microbial inflammatory M1 macrophages while suppressing a tissue-reparative macrophage phenotype. Its effects were protective during enteropathic infection with *Citrobacter rodentium* but resulted in pathological inflammation following mucosal barrier breach. We confirmed that ILC-derived GM-CSF enhanced macrophage microbicidal activity, while suppressing wound-healing transcriptional programs, including collagen and platelet-derived growth factor (PDGF) production, suggesting that some level of GM-CSF signaling may be potentially beneficial in limiting the development of intestinal fibrosis. Indeed, the transcriptional signature of macrophages isolated from ILC-depleted colons was significantly enriched in Crohn's disease biopsies obtained from patients with complicated disease, including those with stricturing disease. GM-CSF also reinforced mucosal type 17 immunity through increased production of ILC3- and Th17 cell-stimulating cytokines by macrophages, setting up a positive feedback loop to promote IL-22 secretion and epithelial AMP production. Together, our data reveal the importance of GM-CSF and ILC-macrophage signaling axis in calibrating macrophage polarization to augment anti-bacterial responses during infection and inflammation, while inhibiting the generation of pro-fibrotic macrophages.

RESULTS

GM-CSF Is an Upstream Regulator of Inflammatory Macrophage Function in the Intestine

To identify candidate cytokines that might promote inflammatory macrophage function in the intestine, we assessed the transcriptional profile of flow-sorted colonic Ly6C^{lo} MHC-II^{+} macrophages (Figure 1A) isolated from healthy and inflamed mouse colon following DSS administration and applied Ingenuity Pathway Analysis (IPA). IPA is a powerful informatic tool that enables an unbiased assessment of phenotype-associated biological pathways in transcriptomic datasets (Dutertre et al., 2019; Mahmoudi et al., 2019). This identified several candidate regulators in inflamed macrophages (Figure S1A). Of these, GM-CSF was notable due to its high enrichment *Z* score and its proximal hierarchical location in the regulatory network, controlling the activation of several key cytokines including tumor necrosis factor (TNF) and IL-1β (Figure 1B). Indeed, predicted GM-CSF-regulated genes in DSS macrophages included those involved in inflammasome activation (*Il1b*, *Hif1a*, *Nlrp3*), prostaglandin E2 production (*Ptgs2*) and co-stimulation (*Cd40*, *Cd80*) (Figure S1B), and GM-CSF receptor subunits (*Csf2ra*, *Csf2rb*, *Csf2rb2*) were highly expressed in murine colonic macrophages (Figure S1C). To determine the relevance of these observations to human intestinal macrophages, we analyzed a recently published single cell RNA sequencing (scRNA-seq) study in ileal Crohn's disease (Martin et al., 2019). Within the mononuclear phagocyte (MNP) cluster (Figure S1D), we identified resident and inflammatory macrophage clusters (Figure 1C) based on canonical marker expression (Figure S1E). Resident macrophages were enriched in uninflamed ileal biopsies, while inflammatory macrophages were almost entirely restricted to inflamed mucosa (Figure 1C). Application of IPA to this inflammatory macrophage dataset also identified GM-CSF as a candidate upstream regulator, with a similar dominant hierarchical position within the interaction network to that observed in DSS macrophages (Figure 1D). Together, these analyses suggest that GM-CSF is a critical modulator of inflammatory macrophage function in intestinal inflammation in humans and mice.

Several leukocyte subsets can produce GM-CSF, including Th17 cells and ILCs (Hirota et al., 2018; Robinette et al., 2015). To identify the major source of colonic GM-CSF, we profiled cytokine production across ILC and T cell subsets in DSS-treated and control C57BL/6 mice (Figures 1E and S1F). ILC3s were the major GM-CSF-producing cell type in the colon (Figure 1F), with lower levels detected in γδ T cells and ILC2s. While the frequency of GM-CSF^{+} cells did not change with DSS administration (Figure S1G), GM-CSF production on a per cell basis was only augmented in ILC3s during intestinal inflammation (Figure 1F), identifying them as the important dynamic source of GM-CSF during inflammation. Similarly, in human intestinal samples, analysis of scRNA-seq data confirmed ILCs as the major cellular source of GM-CSF in the colon in homeostasis (Figure 1G), with augmented transcript levels evident in inflamed ulcerative colitis (Figure 1G).

ILCs and GM-CSF Augment Intestinal Macrophage Activation during Enteropathic Infection and Colitis

To investigate the effect of ILC-derived GM-CSF on intestinal MNP function, we depleted ILCs from *Rag1/2*$^{-/-}$ mice, which lack adaptive immunity thereby avoiding the confounding effects of T cell-derived cytokines. Anti-CD90.2 and control antibody-treated mice were challenged with the enteropathic bacterium *Citrobacter rodentium* or dextran sodium sulfate (DSS), a colitogen that induces epithelial barrier breakdown (Figures S2A and S2B). ILC depletion led to a reduction in disease severity and local inflammation in mice challenged with DSS, as evidenced by lower weight loss, colon weight, and colon shortening (Figures 2A and S2C), as well as improved survival (Figure S2D). Conversely, in response to *C. rodentium* challenge, ILC-depleted mice exhibited a higher local and systemic bacterial burden at day 7 post-infection (Figures 2B and 2C). Despite

CellPress
OPEN ACCESS

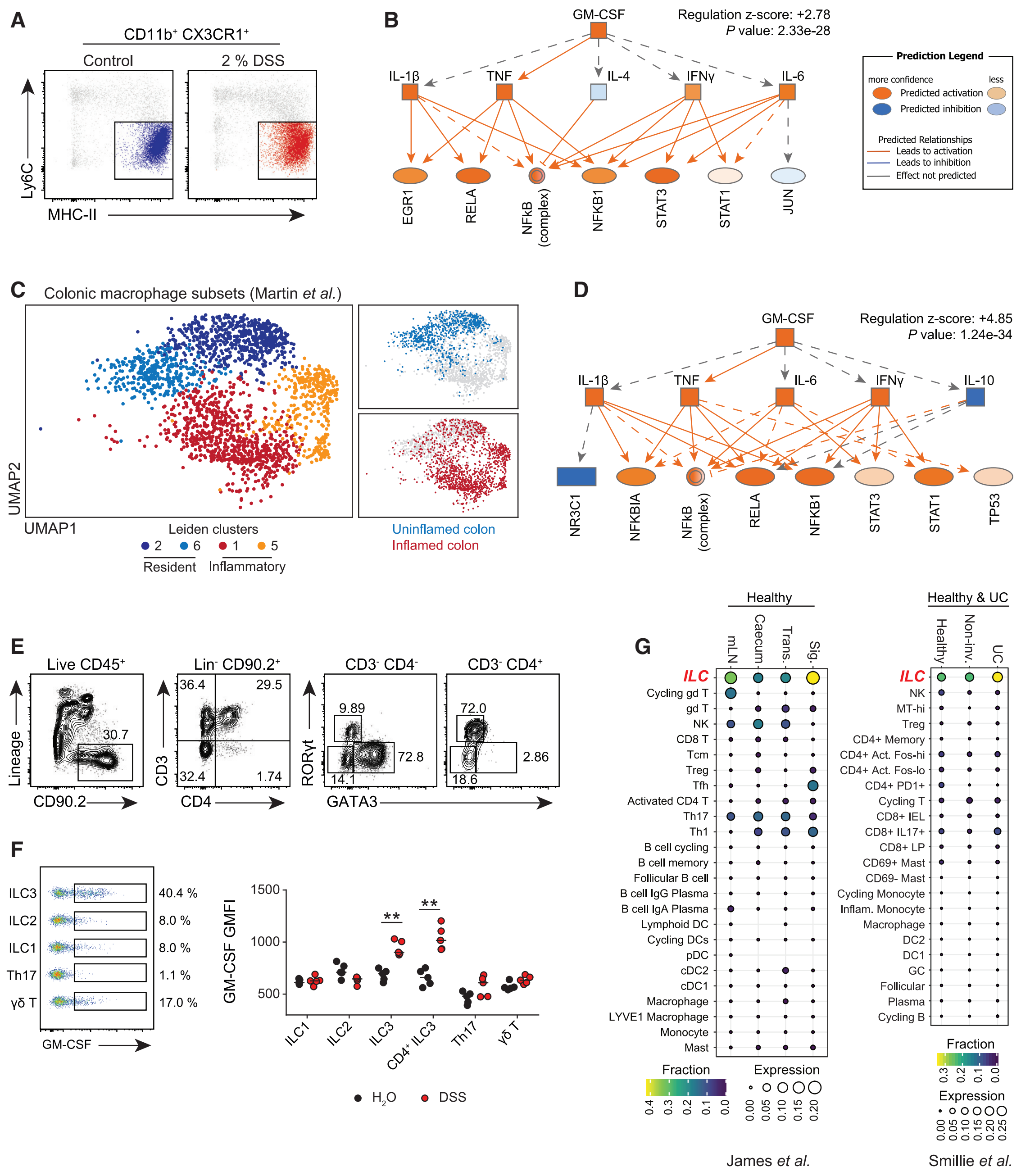

Figure 1. GM-CSF Is an Upstream Regulator of Inflammatory Macrophage Function in the Intestine

(A) Schematic of colonic CX3CR1^{+} CD11b^{+} Ly6C^{lo} MHC-II^{+} macrophages from control or 2% DSS-treated C57BL/6 mice, analyzed by RNA-seq. Data derived from GEO: GSE109040.

(B) GM-CSF-associated upstream regulatory network in DSS macrophages derived by ingenuity pathway analysis (IPA).

(C) UMAP of human ileal resident and inflammatory macrophage subsets from Crohn's disease patients derived by scRNA-seq, showing tissue of origin. Data derived from GEO: GSE134809.

(D) GM-CSF-associated upstream regulatory network in inflammatory macrophages derived by IPA.

(E) Murine colonic ILC and T cell gating strategy.

(F) Intracellular GM-CSF staining for colonic ILC and T cell subsets between control (n = 5) and 2% DSS-treated (n = 5) C57BL/6 mice. Median indicated. Data are representative of two independent experiments.

(G) *CSF2* expression by leukocyte subsets across different human gut tissue sites (ArrayExpress: E-MTAB-8007, E-MTAB-8474), and by colonic leukocyte subsets in ulcerative colitis patients (UC; non-involved UC) and health controls (dataset SCP259) by scRNA-seq.

p values were calculated using IPA software (B and D) or the nonparametric Mann-Whitney U test (F). *$p < 0.05$; **$p < 0.01$; ***$p < 0.001$; ****$p < 0.0001$. See also Figure S1.

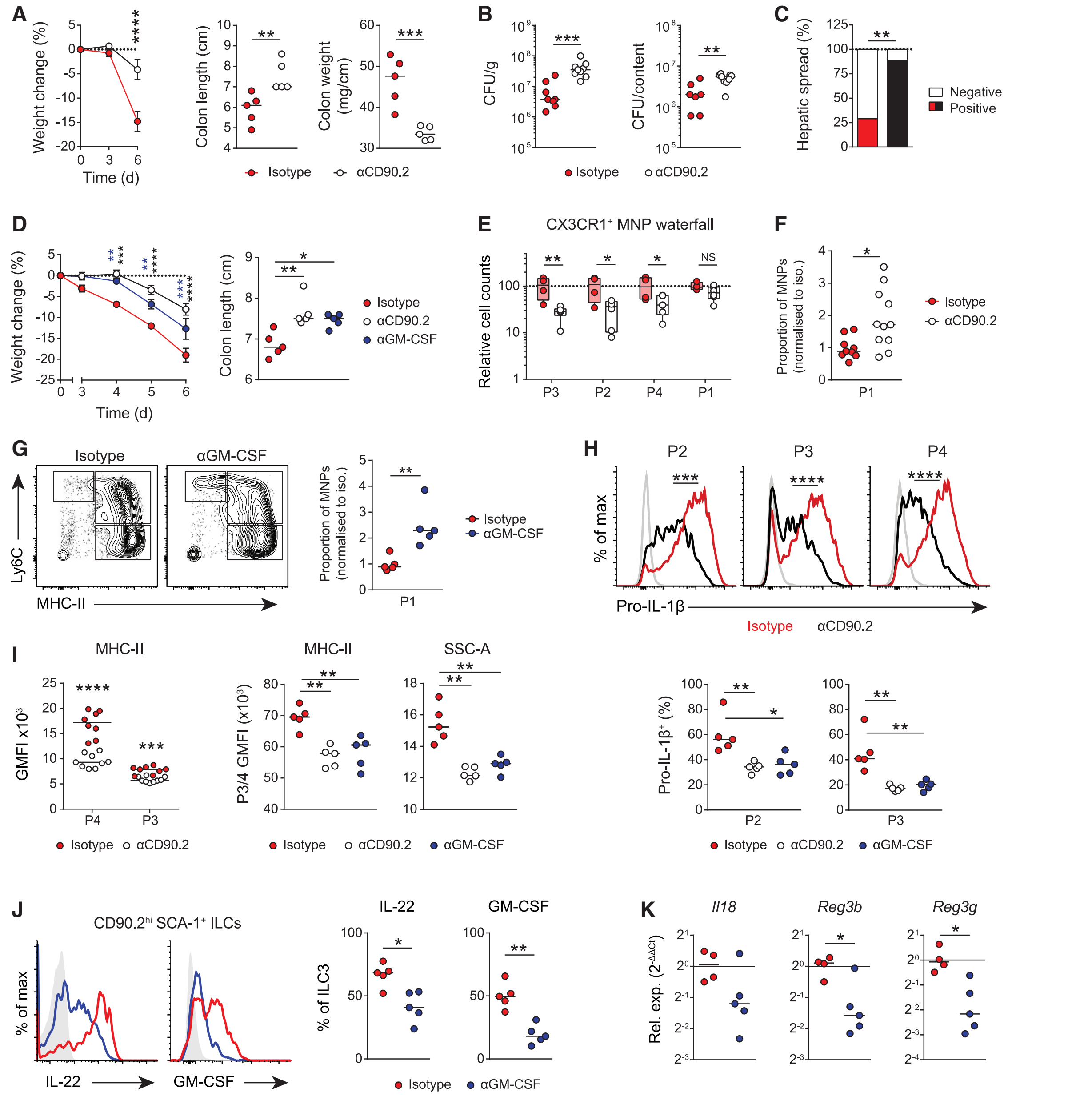

Figure 2. ILCs and Innate GM-CSF Augment Intestinal Macrophage Activation during Enteropathic Infection and Colitis

(A) Weight change (left) and colon clinical parameters (right) in *Rag2*$^{-/-}$ mice treated with 2% DSS ± isotype (n = 5) or anti-CD90.2 IgG (n = 5) antibodies. Mean ± SEM (weight change) and medians (colon parameters) indicated.

(B and C) *C. rodentium* colony-forming units (CFUs) in caecum (B) and liver (C) from isotype (n = 8) or anti-CD90.2 IgG treated (n = 9) *Rag2*$^{-/-}$ mice at 7 days post-infection (dpi). Medians indicated in (B).

(D) Clinical parameters in *Rag2*$^{-/-}$ mice treated as in (A) or anti-GM-CSF IgG antibodies for 6 days. Mean ± SEM (weight change) and medians (colon parameters) indicated. n = 5 per group.

(E) Relative colonic CX3CR1$^+$ MNP counts in mice treated as in (A). Data normalized to isotype. Medians indicated. n = 8–9 per group.

(F and G) Proportion of P1 monocytes of total MNPs, normalized to isotype IgG, from mice treated in (A)–(F) or in (D)–(G). Median indicated. n = 5 per group.

(H) Pro-IL-1β expression by colonic MNP subsets in mice treated as in (A) (top row, n = 8–9 per group) or in (D) (bottom row, n = 5 per group).

(I) MHC-II expression in P3/P4 colonic MNPs in mice treated as in (A) or as in (D). Medians indicated. n = 5–9 per group.

(J) IL-22 and GM-CSF production by CD90.2^{hi} SCA-1$^+$ ILCs in *Rag2*$^{-/-}$ mice treated as in (D). Medians indicated. n = 5 per group.

(K) Colon tissue qPCR of IL-22-dependent epithelium genes in mice treated as in (D).

n = 4–5 per group. Data are representative of three or more independent experiments, with single experiments (A–E, G, J, and K) or two pooled independent experiments (F, H, and I) shown. p values were calculated using a two-way ANOVA with Sidak's multiple comparisons test (A) (for weight loss), the nonparametric Mann-Whitney U test (A) (for colon parameters), in (B) and (D) (for colon parameters), and in (F)–(K), Chi-square test (C), two-way ANOVA with Tukey's multiple comparisons test (D) (weight loss), and multiple t tests with Holm-Sidak correction (E). *p < 0.05; **p < 0.01; ***p < 0.001; ****p < 0.0001. See also Figure S2.

this higher bacterial burden, colon length was also significantly greater in anti-CD90.2 IgG treated mice following *C. rodentium* challenge (Figure S2E), consistent with an attenuated local inflammatory response. Commensurate with a central role for GM-CSF in these clinical phenotypes, neutralization with an anti-GM-CSF antibody improved clinical parameters in DSS-induced colitis to a similar extent to that observed with ILC depletion, including reduced weight loss and increased colon length (Figure 2D). Next, we profiled immune cell infiltrates; intestinal $CX3CR1^+$ macrophages are derived from infiltrating monocytes that progressively lose Ly6C expression and increase MHC-II expression (P1 → P4 subsets) (Tamoutounour et al., 2012; Figure S2F). During colitis, $Ly6C^{lo}$ $MHC\text{-}II^+$ $CX3CR1^{int}$ P3 macrophages are largely derived from newly recruited inflammatory monocytes, while $F4/80^{hi}$ P4 macrophages include a small number of resident macrophages (Bain et al., 2013; Shaw et al., 2018; Zigmond et al., 2012). We observed a reduction in maturing macrophage populations (P2 → P4) following ILC depletion, with little change in monocyte numbers (Figure 2E). Indeed, we observed a relative increase in the proportion of newly infiltrated monocytes (P1) among $CX3CR1^+$ MNPs following DSS challenge (Figure 2F), suggesting defective monocyte maturation in the absence of ILCs. GM-CSF neutralization led to a similar proportional increase in colonic monocytes (Figure 2G). ILC depletion also led to a reduction in colonic neutrophil infiltration, with little change in eosinophil number (Figure S2G). $CX3CR1^+$ MNPs also differed phenotypically following ILC depletion, with a pronounced reduction in pro-IL-1β expression across MNP subsets (Figures 2H and S2H), as well as reduced cellular granularity and MHC-II expression (Figures 2I and S2I), that was phenocopied by GM-CSF neutralization. Importantly, we did not observe GM-CSF expression in $CD90.2^+$ non-hematopoietic cells in the colon (Figure S2J).

IL-1β is a potent activator of ILC3 cytokine production *in vitro* (Figure S2K). Given the profound effect of ILC depletion or GM-CSF neutralization on macrophage activation and the production of IL-1β *in vivo*, we hypothesized that the downstream type 17 response induced by IL-1β may be regulated by GM-CSF through ILC3-MNP crosstalk. *In vitro* ILC3 secretion of IL-22 during co-culture with macrophages was attenuated by GM-CSF neutralization (Figure S2L), despite an absence of GM-CSF receptor expression on intestinal ILC3s (Figure S2M), suggesting that ILC3-derived GM-CSF ultimately amplifies ILC activation via its effects on macrophage cytokine production. *In vivo*, neutralization of GM-CSF in *Rag2*$^{-/-}$ mice subjected to DSS-induced colitis reduced IL-22 and GM-CSF production by $CD90.2^{hi}$ $SCA1^+$ ILC3s (Figure 2J). Furthermore, expression of IL-22-dependent genes produced by the intestinal epithelium were attenuated by GM-CSF neutralization, including RegIIIγ (Figure 2K) demonstrating that optimal ILC3 activation is dependent on GM-CSF-mediated MNP priming via a positive feedback loop.

GM-CSF Drives Inflammatory Macrophage Function and Type 17 Immunity *In Vivo*

Although GM-CSF neutralization phenocopied the clinical and immunological features of ILC depletion *in vivo*, we sought to more closely interrogate whether the principal impact of ILCs on intestinal macrophage function was due to GM-CSF secretion. To address this question, we performed RNA sequencing (RNA-seq) on sorted colonic $Ly6C^{lo}$ $MHC\text{-}II^{hi}$ macrophages from ILC-depleted mice following induction of DSS colitis. We observed widespread changes in gene expression in colonic macrophages in the absence of ILCs (Figure S3A), including downregulation of several important gene networks, most notably IL-12/23-associated innate immune activation (Figure S3B). In line with the observed reduction in colonic macrophage IL-1β production, gene set enrichment analysis (GSEA) also demonstrated a significant reduction in an inflammasome-associated gene set in intestinal macrophages in the absence of ILCs, including *Nlrp3* and *Il1b* (Figure S3C).

IPA of upstream regulators predicted to be attenuated following ILC depletion identified GM-CSF as a central cytokine regulator that was deficient in the absence of ILCs (Figure 3A). Consistent with this, transcription factor (TF) analysis demonstrated a reduction in *Bhlhe40* (recently shown to be a GM-CSF-dependent TF in alveolar macrophages) (Huynh et al., 2018) in intestinal macrophages isolated from ILC-depleted mice (Figure 3B). Furthermore, in the absence of ILCs, there was a significant reduction in other components of the GM-CSF signaling pathway, including *Csf2rb* and *Csf2rb2* and the intracellular signaling molecule *Jak2* (Figure S3D). Indeed, comparison of the transcriptome of macrophages cultured in the presence or absence of GM-CSF for 24 h *in vitro* to that of colonic macrophages isolated following ILC depletion demonstrated that genes that were highly induced by GM-CSF were significantly reduced in colonic macrophages following ILC depletion, with a corresponding increase in genes suppressed by GM-CSF (Figure 3C). Therefore, in the absence of ILCs, intestinal macrophages exhibit a pronounced defect in GM-CSF-associated gene expression consistent with the conclusion that ILCs represent an important source of GM-CSF *in vivo* that drives an inflammatory macrophage phenotype.

While a useful and commonly used model to study ILC function in the absence of cofounding T cell effects, *Rag1/2*$^{-/-}$ mice are known to have a hyperactivated ILC compartment due to the absence of regulatory T cells (Erdman et al., 2003). Therefore, we sought to determine the importance of GM-CSF in driving an inflammatory macrophage phenotype in immune-replete animals by generating bone marrow chimeras with *Csf2rb*$^{-/-}$ bone marrow (Figure S3E). Despite similar immune reconstitution (Figures S3F and S3G), mice receiving 100% *Csf2rb*$^{-/-}$ bone marrow were more susceptible to challenge with *C. rodentium* (Figures 3D and S3H) compared to mice receiving 100% wild-type (WT) bone marrow. As with GM-CSF neutralization, absence of GM-CSF receptor signaling caused an accumulation of immature monocytes (Figure 3E) and a reduction in pro-IL-1β expression by intestinal MNPs (Figure S3I). To determine if the effect of GM-CSF in promoting intestinal macrophage function was cell-intrinsic, we generated mixed *Csf2rb*$^{-/-}$:*Csf2rb*$^{+/+}$ bone marrow chimeras (Figure S3E). Relative to $CD45.1^+$ WT cells, *Csf2rb*$^{-/-}$ $CX3CR1^+$ MNP waterfall subsets were impaired in their maturation from monocytes to $Ly6C^{lo}MHC\text{-}II^{hi}$ macrophages (Figure 3F) and in their ability to produce pro-IL-1β (Figure 3G), with macrophages showing reduced MHC-II expression and granularity (Figure 3H).

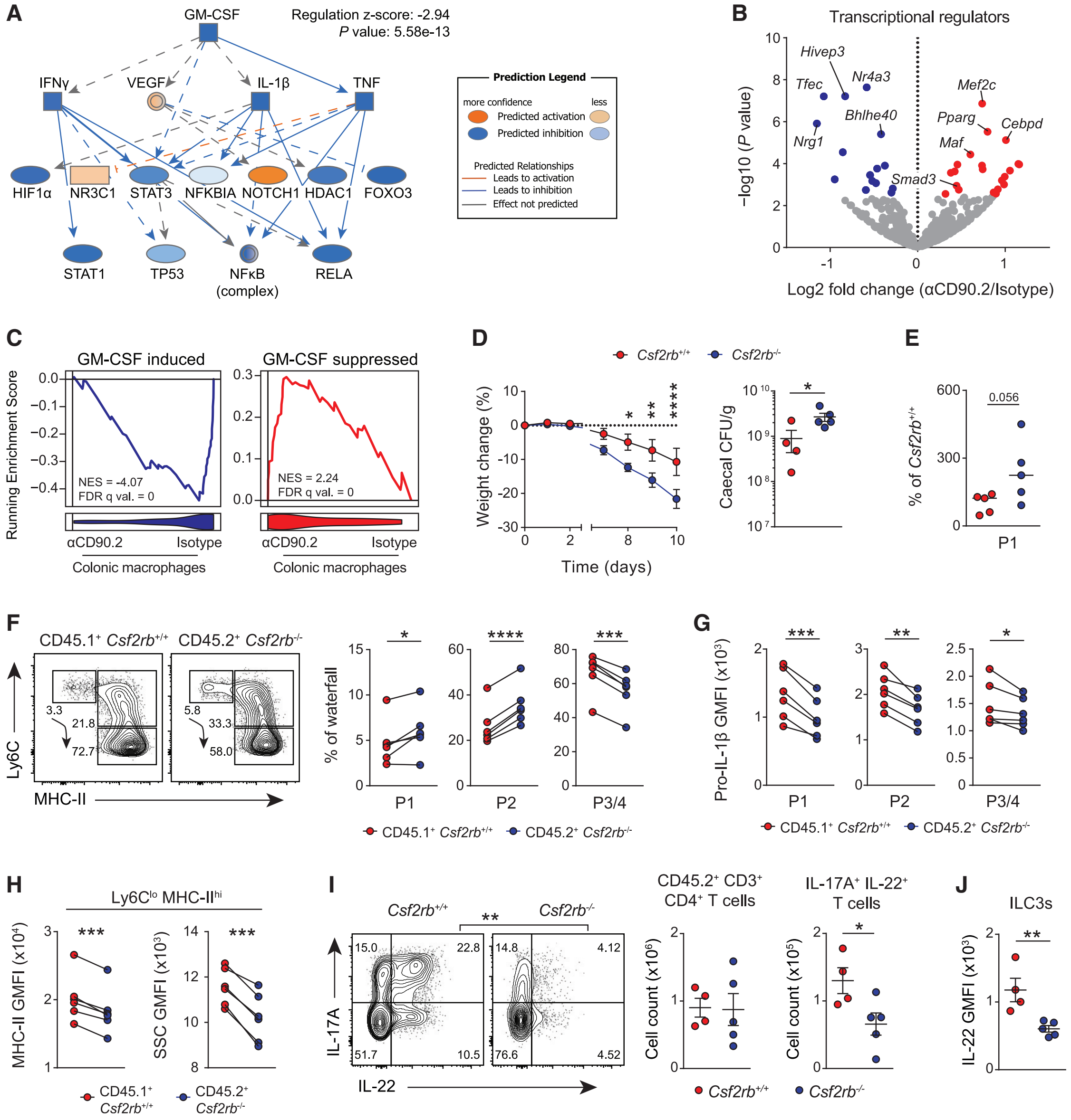

Figure 3. GM-CSF Drives Inflammatory Macrophage Function and Type 17 Immunity *In Vivo*

(A) GM-CSF-associated upstream regulatory network downregulated by anti-CD90.2 IgG treatment, derived by IPA, in colonic Ly6C^{lo} MHC-II^{+} macrophages from *Rag2*$^{-/-}$ mice treated with 2% DSS ± isotype or anti-CD90.2 IgG. n = 6 per group.

(B) RNA-seq volcano plot of transcriptional regulators (GO:0140110) from macrophages in (A).

(C) GSEA of the GM-CSF signatures (top 200 GM-CSF-induced (left) or GM-CSF-suppressed (right) genes in BMDMs) in colonic Ly6C^{lo} MHC-II^{+} macrophages from (A). GM-CSF signatures derived from ArrayExpress: E-MTAB-792.

(D) Weight change (left) and cecal CFU at 10 dpi (right) in 100% *Csf2rb*$^{+/+}$ and 100% *Csf2b*$^{-/-}$ bone marrow chimeras infected with *C. rodentium*. Mean ± SEM indicated. n = 5 per group.

(E) Proportion of P1 monocytes of total CX3CR1^{+} CD11b^{+} MNPs in bone marrow chimeras, normalized to *Csf2rb*$^{+/+}$ mice.

(F and G) Waterfall subset frequency (F) and pro-IL-1β expression (G) of paired *Csf2rb*$^{+/+}$ and *Csf2rb*$^{-/-}$ colonic MNP subsets in 80:20 bone marrow chimeric mice at 7 dpi with *C. rodentium*.

(H) MHC-II expression and side scatter of Ly6C^{lo} MHC-II^{+} macrophages in (F).

(I and J) Type 17 cytokine production by CD4^{+} T cells (I) and ILCs (J) in 100% *Csf2rb*$^{+/+}$ or 100% *Csf2rb*$^{-/-}$ bone marrow chimeric mice at 10 dpi with *C. rodentium*. Mean ± SEM indicated. n = 4–5 per group. Data are representative of two independent experiments. p values were calculated using IPA software (A), standard DESeq 2 method with multiple comparisons correction using the Benjamini-Hochberg procedure (B), two-way ANOVA with Sidak's multiple comparisons test (D) (weight loss), Student's t test (D [CFU analysis], I, and J), Mann-Whitney U test (E), and a paired t test (F–H). *$p < 0.05$; **$p < 0.01$; ***$p < 0.001$; ****$p < 0.0001$. See also Figure S3.

In the absence of macrophage responsiveness to GM-CSF, there was also an attenuation of T cell-derived IL-17A and IL-22 (Figure 3I). Furthermore, IL-22 production, but not IL-17A, by CD4$^-$ CD3$^-$ CD90.2^{hi} ILC3s was also impaired in the absence of GM-CSF receptor signaling (Figures 3J and S3J), with no difference in colonic B cells (Figure S3K).

ILCs and GM-CSF Promote M1 and Suppress M2 Colonic Macrophage Polarization

Hallmarks pathway analysis of our colonic macrophage RNA-seq dataset demonstrated a reduction in a number of canonical inflammatory pathways and in glycolysis genes following ILC depletion, consistent with a pro-inflammatory role for GM-CSF (Figure 4A). However, we also noted an increase in pathways associated with tissue repair and regeneration, including epithelial-mesenchymal transition, myogenesis, and angiogenesis (Figure 4A). Such polarization of macrophage function is reminiscent of pro-inflammatory M1 and anti-inflammatory M2 macrophage phenotypes (Murray et al., 2014). We therefore hypothesized that during intestinal infection and inflammation, the presence of ILCs promotes an M1 phenotype in tissue macrophages while suppressing a wound healing M2-like response. Consistent with this, we observed increased expression of genes associated with M2 macrophage activation, such as *Retnla*, *Pparg*, and *Tgfbr2*, in colonic macrophages of ILC-depleted mice (Figure 4B). Furthermore, alignment of our sorted colonic macrophage dataset with the transcriptional profiles of a spectrum of macrophage activation states (Xue et al., 2014) demonstrated a significant reduction in the M1 LPS-stimulated macrophage signature and an increase in the IL-4 M2 signature following ILC depletion (Figure 4C). Together, these data suggest that ILCs regulate intestinal macrophage polarization, controlling the balance of pro-inflammatory anti-microbial and pro-repair phenotypes that ultimately shapes the course of inflammation.

To determine whether GM-CSF signaling was required for the polarization of intestinal macrophages *in vivo*, we flow-sorted WT and *Csf2rb*$^{-/-}$ intestinal MHC-II$^+$ CD64$^+$ macrophages from mixed bone marrow chimeric mice following challenge with *C. rodentium* and performed qPCR. In addition to pro-IL-1β and MHC-II (Figure 3), *Csf2rb*$^{-/-}$ macrophages had reduced expression of *Il23a*, *Nos2*, and *Slc2a1*, and an increase in *Il10* and *Tgfbr2* expression (Figure 4D), consistent with a reduction in M1 transcriptional programs and an increase in M2 polarization in the absence of GM-CSF *in vivo*.

ILC3s have been implicated in IBD pathogenesis, with increased numbers of ILC3s observed in inflamed intestine in Crohn's disease (CD) in particular (Buonocore et al., 2010; Geremia et al., 2011). To test whether ILC might influence differing macrophage polarization in human IBD, we analyzed mucosal biopsies from patients with ulcerative colitis (UC) and ileal CD, as well as healthy controls. Using cell signatures derived from a recent intestinal scRNA-seq dataset (Smillie et al., 2019), we performed single sample gene set enrichment analysis and demonstrated a significant positive correlation between ILC and M1 macrophage signatures in disease biopsies (Figure 4E). Although correlation does not prove causation, these data mirror our findings in the murine intestine, and are consistent with a role for ILCs in controlling macrophage polarization during human intestinal inflammation. Furthermore, this association was substantially stronger than that of T cell signatures with the M1 macrophage signature (Figure S4A). *CSF2* transcripts also showed a strong positive correlation with M1 macrophage enrichment scores in colonic UC and ileal CD (Figure 4E), higher than other ILC-related cytokines (Figure S4B), supporting the conclusion that ILC3-derived GM-CSF shapes intestinal macrophage polarization in human disease.

ILC3-Derived GM-CSF Drives Macrophage Inflammatory Phenotype

To more directly assess the contribution of ILCs to macrophage polarization *in vivo*, we flow-sorted intestinal RORγt$^+$ ILC3s, the major source of dynamic GM-CSF production in colitis (Figure 1F) and co-cultured them with bone marrow-derived macrophages. The presence of ILC3s (Figure 5A) or ILC3-conditioned media (Figure S5A) enhanced macrophage IL-1β and MHC-II expression, and suppressed IL-10, effects that were inhibited by the addition of a GM-CSF-neutralizing antibody. Similarly, ILC3-conditioned media increased *Tnfsf15* (encoding TL1A), *Il6*, and *Nos2* expression in macrophages, with the increase in *Tnfsf15* and *Il6* being GM-CSF-dependent (Figure S5A). *In vitro*, GM-CSF treatment altered macrophage morphology, increasing surface projections and reducing sphericity (Figures 5B, 5C, and S5B; Videos S1 and S2). Time-lapse imaging demonstrated enhanced macrophage motility and seeks behavior following GM-CSF treatment (Figures 5C and S5B). We observed GM-CSF-dependent increase in phagocytosis of fecal commensals (labeling demonstrated in Figure S5C) in macrophages co-cultured with ILC3s (Figure 5D), consistent with the conclusion that the changes in dynamic behavior increase cellular "search area" and bacterial uptake. Conversely, *in vivo*, macrophages from ILC-depleted colons exhibited an impaired ability to phagocytose commensals, an effect specific to macrophages, with neutrophil phagocytosis unaffected (Figure 5E). Furthermore, colonic MNPs from ILC-depleted mice demonstrated reduced expression of several pattern recognition receptor (PRR)-mediated signaling pathways (Figure S5D). In summary, these data suggest that ILC3-derived GM-CSF augments the inflammatory and microbicidal activity of intestinal macrophages, potentially contributing to the altered systemic spread of bacteria observed with ILC depletion or GM-CSF neutralization.

Given the widespread changes in colonic macrophages, we hypothesized that ILC3s may regulate the metabolic profile of macrophages to support enhanced microbicidal and inflammatory activity. There was a significant reduction in macrophage expression of *Glycolysis* pathway genes in the absence of ILCs (Figure S5E), including *Hk2* and *Ldha* (Figure S5F). Co-culture of macrophages with ILC3s or ILC3-conditioned media was sufficient to induce GM-CSF-dependent expression of glycolytic genes, including *Slc2a1* and *Hk2* (Figures 5F and S5G), and to alter the metabolic phenotype of macrophages (Figure 5G). ILC3 co-culture increased both the extracellular acidification rate (ECAR) and the oxygen consumption rate (OCR) (Figure 5G), consistent with an "energetic" phenotype (Omenetti et al., 2019; Figure 5H). ILC3s increased the glycolytic reserve and capacity

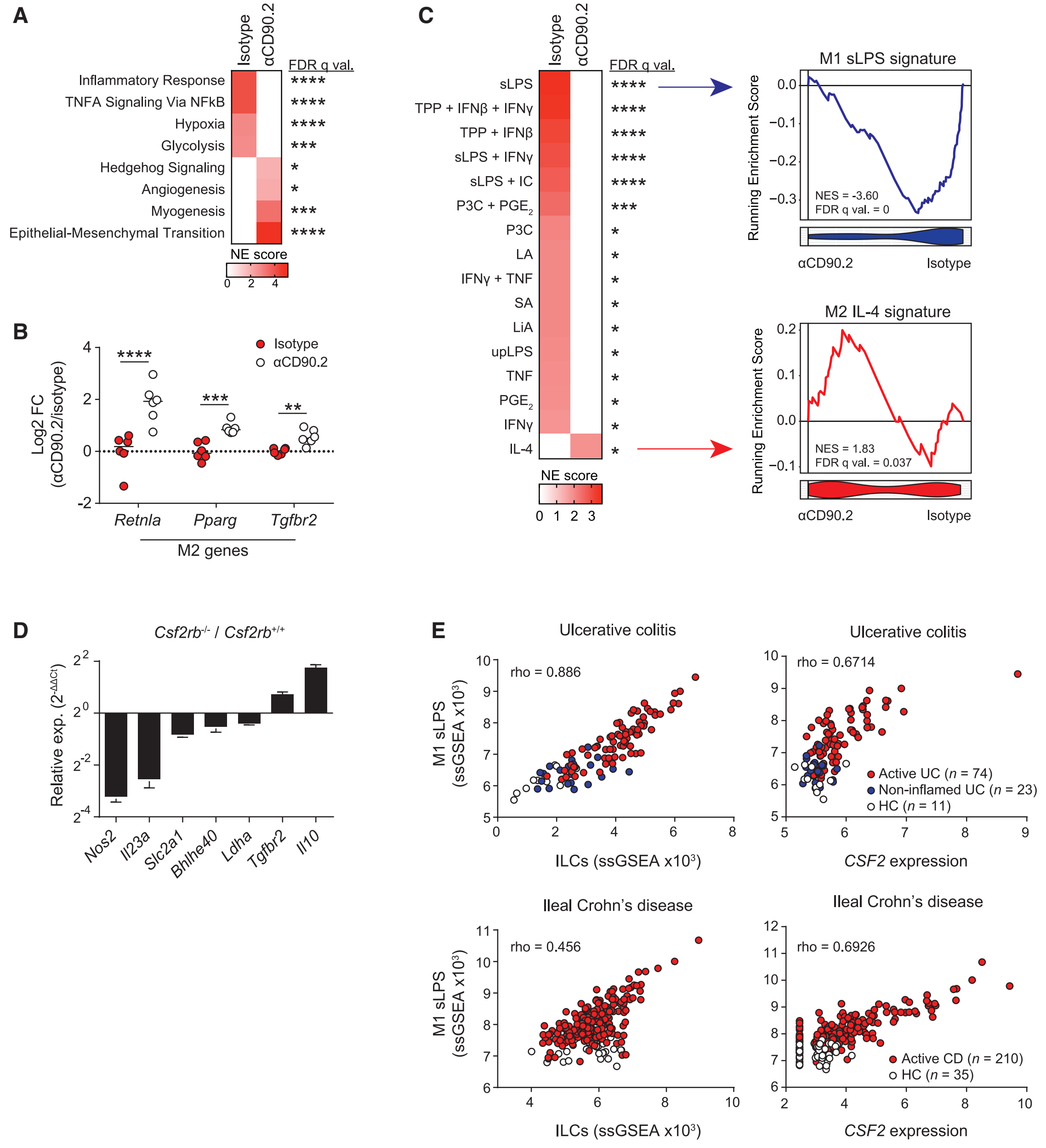

Figure 4. ILCs and GM-CSF Regulate Intestinal Macrophage Polarization

(A) GSEA of top Hallmarks pathways in colonic Ly6C^{lo} MHC-II^{+} MNPs from *Rag2*$^{-/-}$ mice treated with anti-CD90.2 IgG or isotype control and 2% DSS.

(B) Expression of M2 alternative activation genes in colonic MNPs from (A). Medians indicated.

(C) GSEA of top 100 DEGs from human monocyte-derived macrophages (MDMs) treated with various stimuli in colonic MNPs from (A). Data derived from GEO: GSE47189.

(D) Expression of M1 versus M2 genes between flow-sorted intestinal CD45.1^{+} *Csf2rb*$^{+/+}$ and CD45.2^{+} *Csf2rb*$^{-/-}$ Ly6C^{lo} MHC-II^{+} MNPs in 80:20 chimeric mice. Data were derived from pooled Ly6C^{lo} MHC-II^{+} macrophages from 5 mice.

(E) Correlation between single sample GSEA scores of ILC (left) or *CSF2* levels (right) with M1 signatures in ulcerative colitis (healthy control [HC] n = 11, non-inflamed UC n = 23, active UC n = 74) and ileal Crohn's disease biopsies (HC n = 35, ileal CD n = 210). Data derived from GEO: GSE59071 and GEO: GSE93624. p values were calculated using the standard DESeq 2 method with multiple comparisons correction using the BH procedure (B), and Spearman's rank correlation with multiple testing correction (E). *$p < 0.05$; **$p < 0.01$; ***$p < 0.001$; ****$p < 0.0001$.

See also Figure S4.

CellPress
OPEN ACCESS

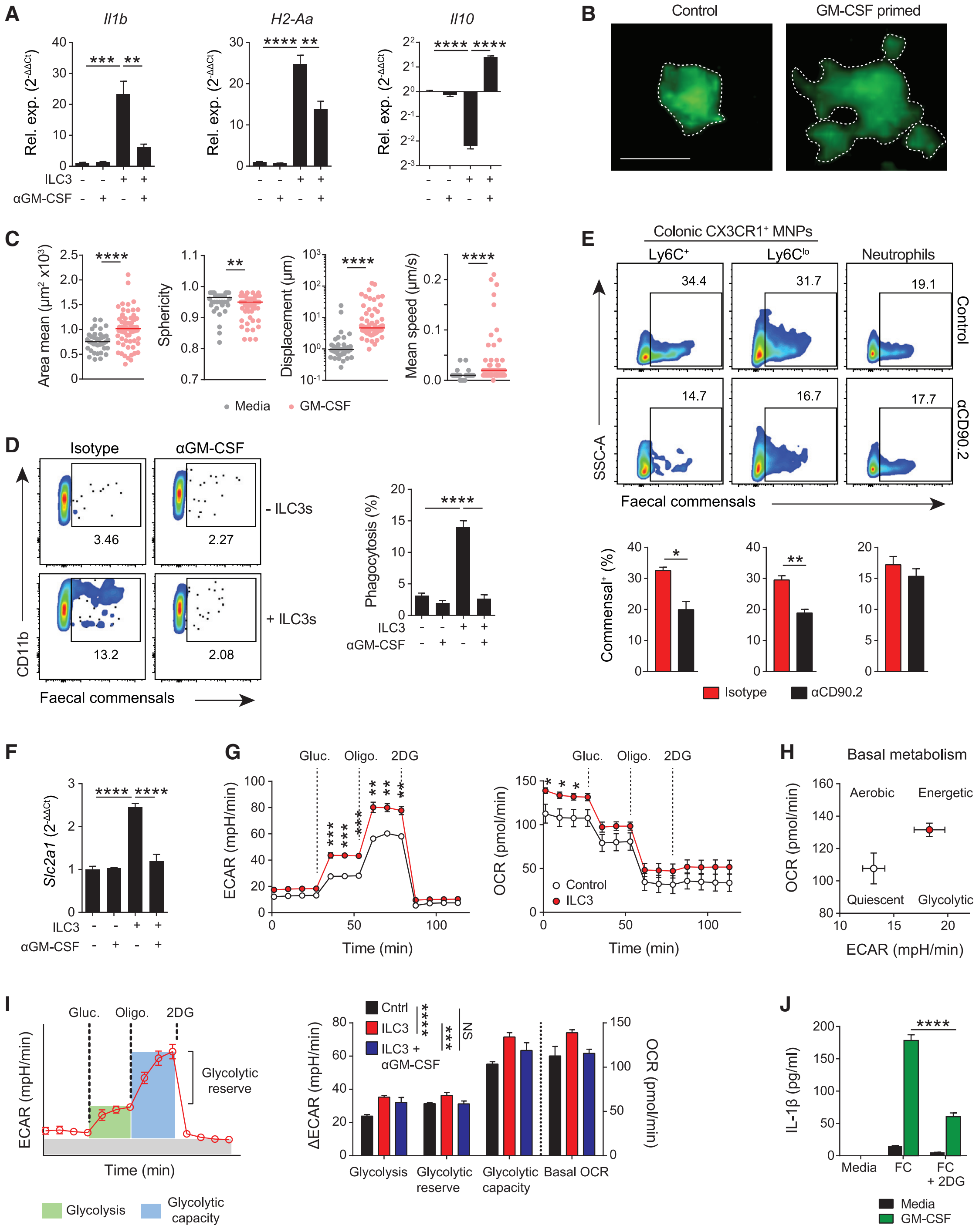

Figure 5. ILC3-Derived GM-CSF Regulates MNP Microbicidal Activity and Metabolism

(A) Cytokine and MHC-II expression in BMDMs cultured with ILC3s ± isotype or anti-GM-CSF IgG for 16 h. Mean ± SEM indicated from triplicates.

(B and C) Morphology and motility analysis of BMDMs after 48 h culture ± control or GM-CSF-supplemented media. Medians are indicated. Scale bar, 10 μm.

(D and E) Phagocytosis of fluorescent commensal microbes by BMDMs co-cultured as in (A) for 72 h (D) or by colonic MNP subsets and neutrophils from mice treated with isotype or anti-CD90.2 IgG at day 6 post 2% DSS administration (E). Mean ± SEM indicated from triplicates.

(F) *Slc2a1* expression in BMDMs co-cultured as in (A). Mean ± SEM indicated from triplicates.

(G) ECAR and OCR for BMDMs primed with ILC3-conditioned media compared to control media for 16 h. Mean ± SEM indicated from 6 measurements.

(H) Basal metabolic profile of BMDMs in (G). Mean ± SEM indicated.

(I) Analysis of features of glycolytic metabolism in BMDMs cultured as in (G) ± anti-GM-CSF IgG. Mean ± SEM indicated from 6 measurements.

(legend continued on next page)

of macrophages, in a GM-CSF-dependent manner (Figures 5I and S5H). This metabolic reprogramming was essential for enhanced macrophage activity, as evidenced by the reduction in GM-CSF-induced IL-1β production in the presence of 2-deoxy-D-glucose (2DG), an inhibitor of glycolysis (Figure 5J).

ILC3s Suppress a Pro-repair Macrophage Phenotype

In addition to the reduction in inflammatory gene networks in macrophages obtained from ILC-depleted mice, we also noted an induction of tissue reparative transcriptional programs (Figure 4A), suggesting that ILC3-derived GM-CSF might actively suppress a pro-repair, wound-healing phenotype in macrophages. Indeed, many of the genes within the epithelial-mesenchymal transition gene set enriched in macrophages isolated from ILC-depleted colons were those required for collagen formation or interaction (Figures 6A and S6A), suggesting that intestinal macrophages may directly contribute to the restoration of the extracellular matrix (ECM) following epithelial barrier breach. Among collagen gene transcripts, *Col4a1* was the most upregulated in macrophages following ILC depletion (Figure 6B), and its expression in BMDM was decreased by the addition of GM-CSF or ILC3 culture supernatants in a GM-CSF-dependent manner (Figure 6C). Notably, collagen synthesis has been demonstrated in macrophages adopting a myofibroblast-like phenotype during tissue and wound repair (Meng et al., 2016; Sinha et al., 2018).

To interrogate if there was evidence for more avid tissue repair beyond macrophages in the absence of ILCs, we assessed transcriptional changes in whole colon samples in DSS colitis (Figure S6B). This confirmed an increase in tissue reparative pathways, including myogenesis and epithelial-mesenchymal transition genes in the absence of ILC (Figure 6D). The intestinal mesenchyme is comprised of a variety of cell types that form the intestinal ECM and connective tissue between the epithelium and muscularis mucosae (Roulis and Flavell, 2016). To determine if there was an increase in any specific mesenchymal cell type in the context of ILC depletion, we curated single cell signatures of all major murine colonic mesenchymal cell types (Kinchen et al., 2018) and assessed the relative enrichment of these cell type signatures in our colonic RNA-seq dataset. This demonstrated a significant enrichment of myofibroblast genes in ILC-depleted colons (Figures 6E and S6C), including *Acta2* that encodes α-SMA (Figure S6D). Myofibroblasts principally arise from fibroblasts and play an important role in wound repair (Tomasek et al., 2002). In addition, fibroblasts and myofibroblasts may also regulate intestinal stem cell activity via the production of Wnt signals (Gregorieff et al., 2005; Lahar et al., 2011; Powell et al., 2011). Notably, we observed an enrichment of Hedgehog signaling and WNT beta catenin signaling pathway genes in ILC-depleted colons (Figure 6D), both of which regulate stem cell fate in intestinal crypts (Fevr et al., 2007; VanDussen et al., 2012).

Macrophages are well known to play a critical role in wound healing by secreting molecules that can activate fibroblasts and myofibroblasts (Wynn and Vannella, 2016), for example, PDGF (Shimokado et al., 1985). We therefore hypothesized that ILC3-derived GM-CSF might not only inhibit a fibroblast/myofibroblast-like phenotype in macrophages, but also their ability to stimulate mesenchymal cells. In keeping with this, colonic macrophages from ILC-depleted mice showed an increase in *Col6a1*, *Col6a2*, and *Col6a3* transcripts (Figure 6B) that together form trimers that make up collagen type VI, previously shown to promote myofibroblast activation in the context of lung fibrosis (Ucero et al., 2019). We also found a marked increase in several growth factor transcripts in colonic macrophages (Figure 6F) and colonic levels of *Pdgfb* were globally elevated in the absence of ILCs (Figure S6E). PDGF-BB has potent pro-wound healing effects, and indeed is licensed for use clinically in the treatment of wounds (Barrientos et al., 2008; Mulder et al., 2009). *In vitro*, macrophage expression of *Pdgfb* was directly suppressed by GM-CSF and by ILC3-conditioned media in a GM-CSF-dependent manner (Figures 6G and S6F). Addition of GM-CSF-treated macrophage conditioned media to fibroblast cultures inhibited the restoration of monolayer integrity following physical damage (Figure 6H, I), while addition of exogenous PDGF-BB promoted this wound-healing response (Figure 6I).

Tissue reparative programs may be helpful to repair barrier breach but if dysregulated may result in fibrosis and intestinal stricturing (Rieder and Fiocchi, 2009). This represents a serious complication of IBD, and is particularly associated with ileal CD (Rieder et al., 2017). We therefore hypothesized that the effect of ILC3-derived GM-CSF on macrophage polarization may be beneficial in reducing progression to intestinal fibrosis. To test this, we examined the transcriptional profile of biopsies from patients with "complicated" CD, comprising those with stricturing or penetrating disease during a 3-year follow-up period post-biopsy (Marigorta et al., 2017). We found that the *epithelial-mesenchymal transition* pathway was the most upregulated in these complicated biopsies compared with "non-complicated" biopsies (Figure S6G). Furthermore, genes upregulated in ILC-depleted macrophages were significantly enriched in CD biopsies obtained from patients with complicated disease (Figure 6J), relative to uncomplicated disease biopsies. This suggests that the magnitude of GM-CSF signaling in macrophages is an important determinant of progression to a stricturing clinical phenotype and is ultimately controlled by ILC3s.

DISCUSSION

GM-CSF has previously been shown to be important for regulating a variety of aspects of intestinal inflammation via its effects on different cell types, including pro-inflammatory and regulatory effects (Griseri et al., 2012, 2015; Mortha et al., 2014; Pearson et al., 2016; Song et al., 2015). Here, we have demonstrated that it plays a critical role in calibrating the phenotype of intestinal macrophages, promoting pro-inflammatory, anti-microbial M1

(J) IL-1β production by BMDMs primed with GM-CSF or control media for 16h before stimulation with fecal commensals (FC) and 2DG for 6 h.
Mean ± SEM indicated from triplicates. Data are representative of two (D, E, and G–J) or three (A, B, and F) independent experiments. p values were calculated using a one-way ANOVA with Tukey's multiple comparisons test (A, D, and F), Mann-Whitney U test (C), Student's t test (E), multiple t tests with Holm-Sidak's multiple comparisons test (G), and two-way ANOVA with Tukey's multiple comparisons test (I and J). $^{*}p < 0.05$; $^{**}p < 0.01$; $^{***}p < 0.001$; $^{****}p < 0.0001$. See also Figure S5.

CellPress
OPEN ACCESS

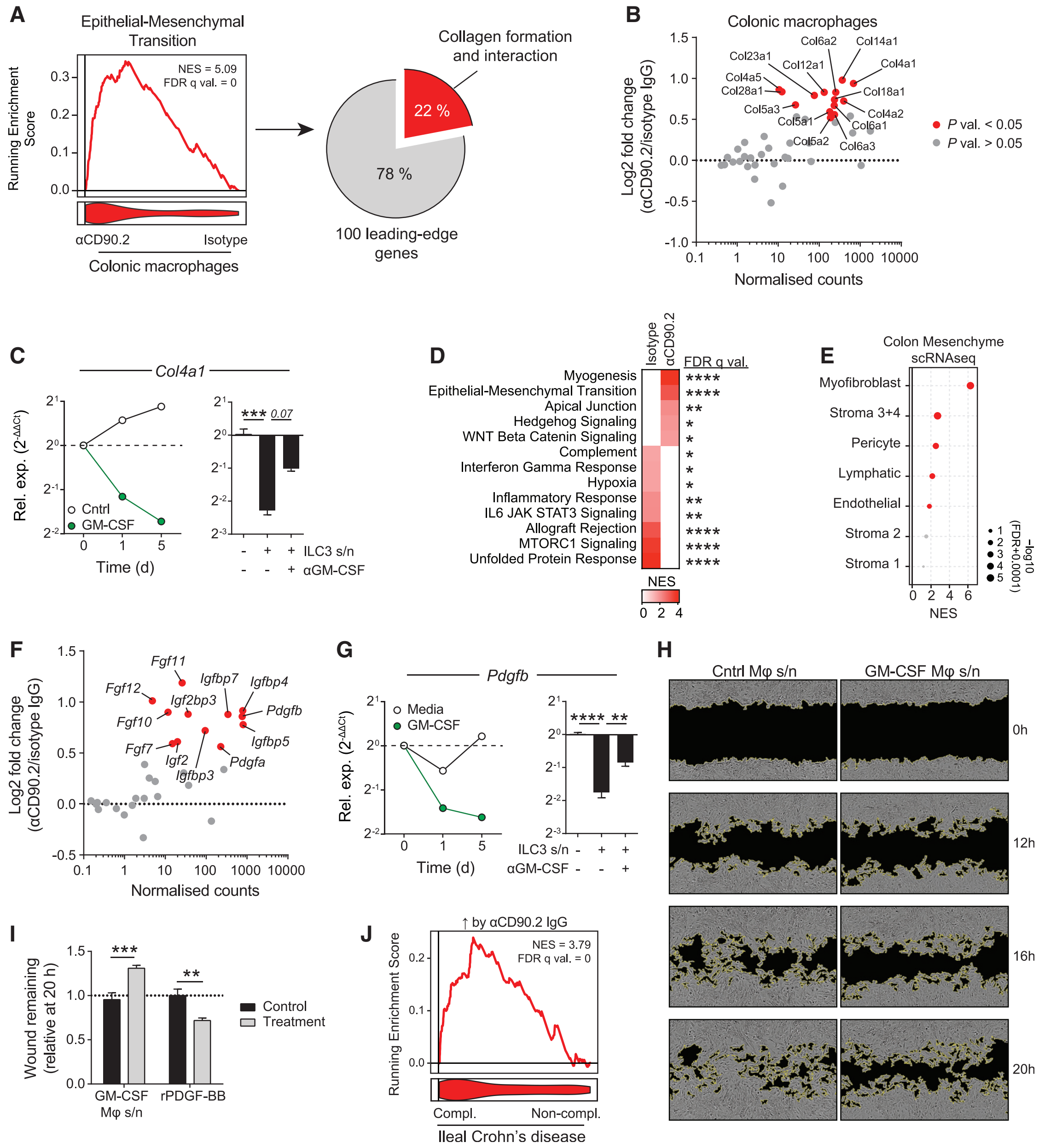

Figure 6. ILCs Suppress a Pro-repair Macrophage Phenotype

(A) GSEA of hallmarks *epithelial-mesenchymal transition* pathway in colonic Ly6C^{lo} MHC-II^{+} MNPs from *Rag2*$^{-/-}$ mice treated with 2% DSS ± isotype or anti-CD90.2 IgG for 6 days, with percentage of leading-edge genes involved in collagen formation/interaction shown. n = 6 per group.
(B) Expression of collagen subunits in colonic MNPs in (A).
(C) *Col4a1* expression by BMDMs primed with GM-CSF or control media (left) or with ILC3-conditioned media ± anti-GM-CSF IgG for 16 h (right). Mean ± SEM indicated from triplicates.
(D and E) RNA-seq GSEA of hallmarks pathways (D) and murine colonic stromal cell signatures (E), derived from murine DSS scRNA-seq dataset GEO: GSE114374, in whole colonic tissue of mice treated as in (A). n = 5 per group.
(F) Growth factor expression in colonic MNPs as in (A). Red dots = p value <0.05.
(G) *Pdgfb* expression in BMDMs treated as in (C). Mean ± SEM indicated from triplicates.
(H and I) Scratch wound assay of murine fibroblasts cultured in conditioned medium from GM-CSF-primed or control BMDMs, or treated with recombinant murine PDGF-BB or control. Mean ± SEM indicated from 5 measurements.
(J) GSEA of the top 200 upregulated genes in colonic Ly6C^{lo} MHC-II^{+} following anti-CD90.2 IgG administration in mucosal biopsies of complicated (n = 27) versus non-complicated (n = 183) ileal Crohn's disease, derived from GEO: GSE93624.

(legend continued on next page)

transcriptional programs while actively suppressing a pro-tissue repair phenotype in the context of ongoing inflammation. Remarkably, depletion of ILCs or neutralization of GM-CSF in the context of enteropathic infection or barrier breach-induced inflammation resulted in enhanced expression of collagen subunits by macrophages, as well as production of fibroblast-stimulating growth factors, including PDGF-BB. Macrophage production of collagen has been previously described in human macrophages *in vitro* and in atherosclerotic plaques (Schnoor et al., 2008; Weitkamp et al., 1999), in mouse models of kidney (Meng et al., 2016) and lung injury (Ucero et al., 2019), and in skin wounds (Sinha et al., 2018), consistent with a transition to a fibroblast/myofibroblast phenotype. Indeed, in the latter study, fate-mapping suggested that two-thirds of granulation tissue fibroblasts in the wound were of myeloid origin (Sinha et al., 2018). Recently, collagen-producing macrophages were shown to directly enhance scar formation *in vivo* in murine and zebrafish models of cardiac fibrosis, in particular through expression of *Col4a1* (Simões et al., 2020). Our study reveals that the tissue milieu during infection or barrier breach provides signals that promote this reparative phenotype in intestinal macrophages, but this is kept in check by local GM-CSF. An excessive wound healing response may result in tissue fibrosis, a well-described complication in IBD. Indeed, strictures represent a major cause of chronic morbidity in CD. Our data raises the possibility that the magnitude of GM-CSF signaling in macrophages is an important determinant of progression to this serious clinical phenotype. In keeping with this, CD patients with detectable GM-CSF autoantibodies have increased frequency of luminal narrowing and strictures (Dykes et al., 2013; Jurickova et al., 2013). Furthermore, impaired GM-CSF bioactivity has been associated with accelerated surgical recurrence in patients with ileal CD (Gathungu et al., 2018) and a frameshift mutation in *CSF2RB* that increases the risk for CD results in reduced monocyte responses to GM-CSF (Chuang et al., 2016). However, the effects of GM-CSF in balancing macrophage polarization, where extremes at either end may result in different types of pathology, inflammation versus stricturing, may explain some of the conflicting data observed with regards to the role of GM-CSF in IBD in humans; recombinant GM-CSF has been used treat CD patients with moderate to severe disease activity and had a high rate of clinical response and remission (Dieckgraefe and Korzenik, 2002; Korzenik et al., 2005) leading to its application to a randomized phase II clinical trial that confirmed superiority to placebo in obtaining a corticosteroid-free clinical remission (Valentine et al., 2009). However, a larger randomized trial found that it was no more effective than placebo for induction of clinical remission or improvement in active CD (Roth et al., 2012). Our work would suggest that recombinant GM-CSF may be more useful in a subset of patients with a stricturing phenotype.

Given the increasing appreciation of macrophage heterogeneity in the intestine (De Schepper et al., 2018; Shaw et al., 2018; Zigmond et al., 2012), further work is required to dissect the role of GM-CSF across macrophage subsets, for example long-lived CD4$^+$ Tim-4$^+$ macrophages and those resident in the muscularis layer, in both health and disease.

In summary, our data reveal that GM-CSF plays a critical role in shaping macrophage metabolism, polarization and function, and that this is largely derived from ILC3s. This ILC3-macrophage crosstalk calibrates macrophage phenotype, balancing pro-inflammatory and pro-wound healing functionality to determine the strength of anti-bacterial responses during infection and inflammation, and susceptibility to fibrosis during tissue repair, with potential therapeutic implications for IBD.

STAR★METHODS

Detailed methods are provided in the online version of this paper and include the following:

- KEY RESOURCES TABLE
- RESOURCE AVAILABILITY
  - Lead Contact
  - Materials Availability
  - Data and Code Availability
- EXPERIMENTAL MODEL AND SUBJECT DETAILS
- METHOD DETAILS
  - DSS-Induced Experimental Colitis
  - Citrobacter rodentium Infection
  - Murine Primary Cell Isolation
  - Flow Cytometry and Cell Sorting
  - Macrophage Cultures and Stimulation
  - Commensal Microbe Phagocytosis
  - Macrophage Commensal Stimulation
  - Macrophage Motility Assay
  - Fibroblast Wound Scratch Assay
  - Metabolic Assays
  - RNA Extraction and Reverse Transcription
  - Quantitative Polymerase Chain Reaction
  - ELISA
  - RNaseq Sample Preparation
  - RNA Sequencing and Analysis
  - Public RNaseq and Microarray
  - Single Cell RNaseq
  - Ingenuity Pathway Analysis
- QUANTIFICATION AND STATISTICAL ANALYSIS

SUPPLEMENTAL INFORMATION

Supplemental Information can be found online at https://doi.org/10.1016/j.celrep.2020.107857.

ACKNOWLEDGMENTS

The authors would like to thank Gordon Dougan (University of Cambridge) for useful discussion and Fiona Powrie (Oxford, UK) for *Csf2rb*$^{-/-}$ mice. The Clatworthy Lab is based in the University of Cambridge Molecular Immunity Unit in the MRC Laboratory of Molecular Biology and is grateful for the use of the core facilities. T.C.-D. and A.F. were supported by University of Cambridge/Wellcome Trust Infection, Immunity and Inflammation PhD Fellowships (102163/

Data are representative of two (H and I) or three (C and G) independent experiments. p values were calculated using standard DESeq 2 method (RNA-seq) or limma (microarray) with multiple comparisons correction using the BH correction (B and F), one-way ANOVA with Tukey's multiple comparisons test (C and G), and multiple t tests with Holm-Sidak's multiple comparisons test (I). *$p < 0.05$; **$p < 0.01$; ***$p < 0.001$; ****$p < 0.0001$. See also Figure S6.

B/13/Z). C.J. was funded by the China Scholarship Council (201408060241). J.R.F. and M.R.C. are supported by the National Institute of Health Research (NIHR) Cambridge Biomedical Research Centre and the NIHR Blood and Transplant Research Unit. T.W.D. and M.R.C. are supported by a Medical Research Council New Investigator Research Grant (MR/N024907/1), a Chan-Zuckerberg Initiative Human Cell Atlas Technology Development Grant, a Versus Arthritis Cure Challenge Research Grant (21777), and an NIHR Research Professorship (RP-2017-08-ST2-002).

AUTHOR CONTRIBUTIONS

T.C.-D. and M.R.C. designed experiments and analyzed data, wrote the manuscript, and conceived the study. T.C.-D., T.W.D., A.F., J.R.F., B.J.S., Z.C., Z.K.T., C.J., L.S.C.L., A.P., and R.J.M. performed experiments and analyzed data. S.C. designed and performed experiments and analyzed data.

DECLARATION OF INTERESTS

The authors declare no competing interests.

Received: October 24, 2019
Revised: April 26, 2020
Accepted: June 12, 2020
Published: July 7, 2020

REFERENCES

Bain, C.C., Scott, C.L., Uronen-Hansson, H., Gudjonsson, S., Jansson, O., Grip, O., Guilliams, M., Malissen, B., Agace, W.W., and Mowat, M. (2013). Resident and pro-inflammatory macrophages in the colon represent alternative context-dependent fates of the same Ly6Chi monocyte precursors. Mucosal Immunol. *6*, 498–510.

Bain, C.C., Bravo-Blas, A., Scott, C.L., Perdiguero, E.G., Geissmann, F., Henri, S., Malissen, B., Osborne, L.C., Artis, D., and Mowat, A.M. (2014). Constant replenishment from circulating monocytes maintains the macrophage pool in the intestine of adult mice. Nat. Immunol. *15*, 929–937.

Barrientos, S., Stojadinovic, O., Golinko, M.S., Brem, H., and Tomic-Canic, M. (2008). Growth factors and cytokines in wound healing. Wound Repair Regen. *16*, 585–601.

Bernshtein, B., Curato, C., Ioannou, M., Thaiss, C.A., Gross-Vered, M., Kolesnikov, M., Wang, Q., David, E., Chappell-Maor, L., Harmelin, A., et al. (2019). IL-23–producing IL-10Rα–deficient gut macrophages elicit an IL-22–driven proinflammatory epithelial cell response. Sci. Immunol. *4*, eaau6571.

Buonocore, S., Ahern, P.P., Uhlig, H.H., Ivanov, I.I., Littman, D.R., Maloy, K.J., and Powrie, F. (2010). Innate lymphoid cells drive interleukin-23-dependent innate intestinal pathology. Nature *464*, 1371–1375.

Castro-Dopico, T., Dennison, T.W., Ferdinand, J.R., Mathews, R.J., Fleming, A., Clift, D., Stewart, B.J., Jing, C., Strongili, K., Labzin, L.I., et al. (2019). Anti-commensal IgG Drives Intestinal Inflammation and Type 17 Immunity in Ulcerative Colitis. Immunity *50*, 1099–1114.

Cella, M., Fuchs, A., Vermi, W., Facchetti, F., Otero, K., Lennerz, J.K., Doherty, J.M., Mills, J.C., and Colonna, M. (2009). A human natural killer cell subset provides an innate source of IL-22 for mucosal immunity. Nature *457*, 722–725.

Chuang, L.S., Villaverde, N., Hui, K.Y., Mortha, A., Rahman, A., Levine, A.P., Haritunians, T., Evelyn Ng, S.M., Zhang, W., Hsu, N.Y., et al. (2016). A Frameshift in CSF2RB Predominant Among Ashkenazi Jews Increases Risk for Crohn's Disease and Reduces Monocyte Signaling via GM-CSF. Gastroenterology *151*, 710–723.

Coccia, M., Harrison, O.J., Schiering, C., Asquith, M.J., Becher, B., Powrie, F., and Maloy, K.J. (2012). IL-1β mediates chronic intestinal inflammation by promoting the accumulation of IL-17A secreting innate lymphoid cells and CD4(+) Th17 cells. J. Exp. Med. *209*, 1595–1609.

Cummings, R.J., Barbet, G., Bongers, G., Hartmann, B.M., Gettler, K., Muniz, L., Furtado, G.C., Cho, J., Lira, S.A., and Blander, J.M. (2016). Different tissue phagocytes sample apoptotic cells to direct distinct homeostasis programs. Nature *539*, 565–569.

De Schepper, S., Verheijden, S., Aguilera-Lizarraga, J., Viola, M.F., Boesmans, W., Stakenborg, N., Voytyuk, I., Schmidt, I., Boeckx, B., Dierckx de Casterlé, I., et al. (2018). Self-Maintaining Gut Macrophages Are Essential for Intestinal Homeostasis. Cell *175*, 400–415.

Dieckgraefe, B.K., and Korzenik, J.R. (2002). Treatment of active Crohn's disease with recombinant human granulocyte-macrophage colony-stimulating factor. Lancet *360*, 1478–1480.

Dutertre, C.-A., Becht, E., Irac, S.E., Khalilnezhad, A., Narang, V., Khalilnezhad, S., Ng, P.Y., van den Hoogen, L.L., Leong, J.Y., Lee, B., et al. (2019). Single-Cell Analysis of Human Mononuclear Phagocytes Reveals Subset-Defining Markers and Identifies Circulating Inflammatory Dendritic Cells. Immunity *51*, 573–589.

Dykes, D.M., Towbin, A.J., Bonkowski, E., Chalk, C., Bezold, R., Lake, K., Kim, M.O., Heubi, J.E., Trapnell, B.C., Podberesky, D.J., and Denson, L.A. (2013). Increased prevalence of luminal narrowing and stricturing identified by enterography in pediatric Crohn's disease patients with elevated granulocyte-macrophage colony stimulating factor autoantibodies. Inflamm. Bowel Dis. *19*, 2146–2154.

Erdman, S.E., Poutahidis, T., Tomczak, M., Rogers, A.B., Cormier, K., Plank, B., Horwitz, B.H., and Fox, J.G. (2003). CD4+ CD25+ regulatory T lymphocytes inhibit microbially induced colon cancer in Rag2-deficient mice. Am. J. Pathol. *162*, 691–702.

Fevr, T., Robine, S., Louvard, D., and Huelsken, J. (2007). Wnt/beta-catenin is essential for intestinal homeostasis and maintenance of intestinal stem cells. Mol. Cell. Biol. *27*, 7551–7559.

Gathungu, G., Zhang, Y., Tian, X., Bonkowski, E., Rowehl, L., Krumsiek, J., Nix, B., Chalk, C., Trapnell, B., Zhu, W., et al. (2018). Impaired granulocyte-macrophage colony-stimulating factor bioactivity accelerates surgical recurrence in ileal Crohn's disease. World J. Gastroenterol. *24*, 623–630.

Geremia, A., Arancibia-Cárcamo, C.V., Fleming, M.P., Rust, N., Singh, B., Mortensen, N.J., Travis, S.P., and Powrie, F. (2011). IL-23-responsive innate lymphoid cells are increased in inflammatory bowel disease. J. Exp. Med. *208*, 1127–1133.

Gregorieff, A., Pinto, D., Begthel, H., Destrée, O., Kielman, M., and Clevers, H. (2005). Expression pattern of Wnt signaling components in the adult intestine. Gastroenterology *129*, 626–638.

Griseri, T., McKenzie, B.S., Schiering, C., and Powrie, F. (2012). Dysregulated hematopoietic stem and progenitor cell activity promotes interleukin-23-driven chronic intestinal inflammation. Immunity *37*, 1116–1129.

Griseri, T., Arnold, I.C., Pearson, C., Krausgruber, T., Schiering, C., Franchini, F., Schulthess, J., McKenzie, B.S., Crocker, P.R., and Powrie, F. (2015). Granulocyte Macrophage Colony-Stimulating Factor-Activated Eosinophils Promote Interleukin-23 Driven Chronic Colitis. Immunity *43*, 187–199.

Hirota, K., Hashimoto, M., Ito, Y., Matsuura, M., Ito, H., Tanaka, M., Watanabe, H., Kondoh, G., Tanaka, A., Yasuda, K., et al. (2018). Autoimmune Th17 Cells Induced Synovial Stromal and Innate Lymphoid Cell Secretion of the Cytokine GM-CSF to Initiate and Augment Autoimmune Arthritis. Immunity *48*, 1220–1232.

Huynh, J.P., Lin, C.C., Kimmey, J.M., Jarjour, N.N., Schwarzkopf, E.A., Bradstreet, T.R., Shchukina, I., Shpynov, O., Weaver, C.T., Taneja, R., et al. (2018). Bhlhe40 is an essential repressor of IL-10 during *Mycobacterium tuberculosis* infection. J. Exp. Med. *215*, 1823–1838.

Ip, W.K.E., Hoshi, N., Shouval, D.S., Snapper, S., and Medzhitov, R. (2017). Anti-inflammatory effect of IL-10 mediated by metabolic reprogramming of macrophages. Science *356*, 513–519.

James, K.R., Gomes, T., Elmentaite, R., Kumar, N., Gulliver, E.L., King, H.W., Stares, M.D., Bareham, B.R., Ferdinand, J.R., Petrova, V.N., et al. (2020). Distinct microbial and immune niches of the human colon. Nat. Immunol. *21*, 343–353.

Joeris, T., Müller-Luda, K., Agace, W.W., and Mowat, A.M. (2017). Diversity and functions of intestinal mononuclear phagocytes. Mucosal Immunol. *10*, 845–864.

Jurickova, I., Collins, M.H., Chalk, C., Seese, A., Bezold, R., Lake, K., von Allmen, D., Frischer, J.S., Falcone, R.A., Trapnell, B.C., and Denson, L.A. (2013). Paediatric Crohn disease patients with stricturing behaviour exhibit ileal granulocyte-macrophage colony-stimulating factor (GM-CSF) autoantibody production and reduced neutrophil bacterial killing and GM-CSF bioactivity. Clin. Exp. Immunol. *172*, 455–465.

Kaser, A., Zeissig, S., and Blumberg, R.S. (2010). Inflammatory bowel disease. Annu. Rev. Immunol. *28*, 573–621.

Kinchen, J., Chen, H.H., Parikh, K., Antanaviciute, A., Jagielowicz, M., Fawkner-Corbett, D., Ashley, N., Cubitt, L., Mellado-Gomez, E., Attar, M., et al. (2018). Structural Remodeling of the Human Colonic Mesenchyme in Inflammatory Bowel Disease. Cell *175*, 372–386.

Korzenik, J.R., Dieckgraefe, B.K., Valentine, J.F., Hausman, D.F., and Gilbert, M.J.; Sargramostim in Crohn's Disease Study Group (2005). Sargramostim for active Crohn's disease. N. Engl. J. Med. *352*, 2193–2201.

Lacey, D.C., Achuthan, A., Fleetwood, A.J., Dinh, H., Roiniotis, J., Scholz, G.M., Chang, M.W., Beckman, S.K., Cook, A.D., and Hamilton, J.A. (2012). Defining GM-CSF- and macrophage-CSF-dependent macrophage responses by in vitro models. J. Immunol. *188*, 5752–5765.

Lahar, N., Lei, N.Y., Wang, J., Jabaji, Z., Tung, S.C., Joshi, V., Lewis, M., Stelzner, M., Martín, M.G., and Dunn, J.C. (2011). Intestinal subepithelial myofibroblasts support in vitro and in vivo growth of human small intestinal epithelium. PLoS ONE *6*, e26898.

Longman, R.S., Diehl, G.E., Victorio, D.A., Huh, J.R., Galan, C., Miraldi, E.R., Swaminath, A., Bonneau, R., Scherl, E.J., and Littman, D.R. (2014). CX_3CR1^+ mononuclear phagocytes support colitis-associated innate lymphoid cell production of IL-22. J. Exp. Med. *211*, 1571–1583.

Mahmoudi, S., Mancini, E., Xu, L., Moore, A., Jahanbani, F., Hebestreit, K., Srinivasan, R., Li, X., Devarajan, K., Prélot, L., et al. (2019). Heterogeneity in old fibroblasts is linked to variability in reprogramming and wound healing. Nature *574*, 553–558.

Manta, C., Heupel, E., Radulovic, K., Rossini, V., Garbi, N., Riedel, C.U., and Niess, J.H. (2013). CX(3)CR1(+) macrophages support IL-22 production by innate lymphoid cells during infection with Citrobacter rodentium. Mucosal Immunol. *6*, 177–188.

Marigorta, U.M., Denson, L.A., Hyams, J.S., Mondal, K., Prince, J., Walters, T.D., Griffiths, A., Noe, J.D., Crandall, W.V., Rosh, J.R., et al. (2017). Transcriptional risk scores link GWAS to eQTLs and predict complications in Crohn's disease. Nat. Genet. *49*, 1517–1521.

Martin, J.C., Chang, C., Boschetti, G., Ungaro, R., Giri, M., Grout, J.A., Gettler, K., Chuang, L.S., Nayar, S., Greenstein, A.J., et al. (2019). Single-Cell Analysis of Crohn's Disease Lesions Identifies a Pathogenic Cellular Module Associated with Resistance to Anti-TNF Therapy. Cell *178*, 1493–1508.

Meng, X.M., Wang, S., Huang, X.R., Yang, C., Xiao, J., Zhang, Y., To, K.F., Nikolic-Paterson, D.J., and Lan, H.Y. (2016). Inflammatory macrophages can transdifferentiate into myofibroblasts during renal fibrosis. Cell Death Dis. *7*, e2495.

Mortha, A., Chudnovskiy, A., Hashimoto, D., Bogunovic, M., Spencer, S.P., Belkaid, Y., and Merad, M. (2014). Microbiota-dependent crosstalk between macrophages and ILC3 promotes intestinal homeostasis. Science *343*, 1249288.

Mulder, G., Tallis, A.J., Marshall, V.T., Mozingo, D., Phillips, L., Pierce, G.F., Chandler, L.A., and Sosnowski, B.K. (2009). Treatment of nonhealing diabetic foot ulcers with a platelet-derived growth factor gene-activated matrix (GAM501): results of a phase 1/2 trial. Wound Repair Regen. *17*, 772–779.

Murray, P.J., Allen, J.E., Biswas, S.K., Fisher, E.A., Gilroy, D.W., Goerdt, S., Gordon, S., Hamilton, J.A., Ivashkiv, L.B., Lawrence, T., et al. (2014). Macrophage activation and polarization: nomenclature and experimental guidelines. Immunity *41*, 14–20.

Omenetti, S., Bussi, C., Metidji, A., Iseppon, A., Lee, S., Tolaini, M., Li, Y., Kelly, G., Chakravarty, P., Shoaie, S., et al. (2019). The Intestine Harbors Functionally Distinct Homeostatic Tissue-Resident and Inflammatory Th17 Cells. Immunity *51*, 77–89.

Pearson, C., Thornton, E.E., McKenzie, B., Schaupp, A.L., Huskens, N., Griseri, T., West, N., Tung, S., Seddon, B.P., Uhlig, H.H., and Powrie, F. (2016). ILC3 GM-CSF production and mobilisation orchestrate acute intestinal inflammation. eLife *5*, e10066.

Polański, K., Young, M.D., Miao, Z., Meyer, K.B., Teichmann, S.A., and Park, J.-E. (2020). BBKNN: fast batch alignment of single cell transcriptomes. Bioinformatics *36*, 964–965.

Popescu, D.M., Botting, R.A., Stephenson, E., Green, K., Webb, S., Jardine, L., Calderbank, E.F., Polanski, K., Goh, I., Efremova, M., et al. (2019). Decoding human fetal liver haematopoiesis. Nature *574*, 365–371.

Powell, D.W., Pinchuk, I.V., Saada, J.I., Chen, X., and Mifflin, R.C. (2011). Mesenchymal cells of the intestinal lamina propria. Annu. Rev. Physiol. *73*, 213–237.

Rieder, F., and Fiocchi, C. (2009). Intestinal fibrosis in IBD–a dynamic, multifactorial process. Nat. Rev. Gastroenterol. Hepatol. *6*, 228–235.

Rieder, F., Fiocchi, C., and Rogler, G. (2017). Mechanisms, Management, and Treatment of Fibrosis in Patients With Inflammatory Bowel Diseases. Gastroenterology *152*, 340–350.

Robinette, M.L., Fuchs, A., Cortez, V.S., Lee, J.S., Wang, Y., Durum, S.K., Gilfillan, S., and Colonna, M.; Immunological Genome Consortium (2015). Transcriptional programs define molecular characteristics of innate lymphoid cell classes and subsets. Nat. Immunol. *16*, 306–317.

Roth, L., MacDonald, J.K., McDonald, J.W., and Chande, N. (2012). Sargramostim (GM-CSF) for induction of remission in Crohn's disease: a cochrane inflammatory bowel disease and functional bowel disorders systematic review of randomized trials. Inflamm. Bowel Dis. *18*, 1333–1339.

Roulis, M., and Flavell, R.A. (2016). Fibroblasts and myofibroblasts of the intestinal lamina propria in physiology and disease. Differentiation *92*, 116–131.

Satoh-Takayama, N., Vosshenrich, C.A., Lesjean-Pottier, S., Sawa, S., Lochner, M., Rattis, F., Mention, J.J., Thiam, K., Cerf-Bensussan, N., Mandelboim, O., et al. (2008). Microbial flora drives interleukin 22 production in intestinal NKp46+ cells that provide innate mucosal immune defense. Immunity *29*, 958–970.

Schnoor, M., Cullen, P., Lorkowski, J., Stolle, K., Robenek, H., Troyer, D., Rauterberg, J., and Lorkowski, S. (2008). Production of type VI collagen by human macrophages: a new dimension in macrophage functional heterogeneity. J. Immunol. *180*, 5707–5719.

Seo, S.-U., Kamada, N., Muñoz-Planillo, R., Kim, Y.-G., Kim, D., Koizumi, Y., Hasegawa, M., Himpsl, S.D., Browne, H.P., Lawley, T.D., et al. (2015a). Distinct Commensals Induce Interleukin-1β via NLRP3 Inflammasome in Inflammatory Monocytes to Promote Intestinal Inflammation in Response to Injury. Immunity *42*, 744–755.

Seo, S.-U., Kuffa, P., Kitamoto, S., Nagao-Kitamoto, H., Rousseau, J., Kim, Y.-G., Núñez, G., and Kamada, N. (2015b). Intestinal macrophages arising from CCR2(+) monocytes control pathogen infection by activating innate lymphoid cells. Nat. Commun. *6*, 8010.

Shaw, T.N., Houston, S.A., Wemyss, K., Bridgeman, H.M., Barbera, T.A., Zangerle-Murray, T., Strangward, P., Ridley, A.J.L., Wang, P., Tamoutounour, S., et al. (2018). Tissue-resident macrophages in the intestine are long lived and defined by Tim-4 and CD4 expression. J. Exp. Med. *215*, 1507–1518.

Shimokado, K., Raines, E.W., Madtes, D.K., Barrett, T.B., Benditt, E.P., and Ross, R. (1985). A significant part of macrophage-derived growth factor consists of at least two forms of PDGF. Cell *43*, 277–286.

Simões, F.C., Cahill, T.J., Kenyon, A., Gavriouchkina, D., Vieira, J.M., Sun, X., Pezzolla, D., Ravaud, C., Masmanian, E., Weinberger, M., et al. (2020). Macrophages directly contribute collagen to scar formation during zebrafish heart regeneration and mouse heart repair. Nat. Commun. *11*, 600.

CellPress
OPEN ACCESS

Sinha, M., Sen, C.K., Singh, K., Das, A., Ghatak, S., Rhea, B., Blackstone, B., Powell, H.M., Khanna, S., and Roy, S. (2018). Direct conversion of injury-site myeloid cells to fibroblast-like cells of granulation tissue. Nat. Commun. *9*, 936.

Smillie, C.S., Biton, M., Ordovas-Montanes, J., Sullivan, K.M., Burgin, G., Graham, D.B., Herbst, R.H., Rogel, N., Slyper, M., Waldman, J., et al. (2019). Intra- and Inter-cellular Rewiring of the Human Colon during Ulcerative Colitis. Cell *178*, 714–730.

Song, C., Lee, J.S., Gilfillan, S., Robinette, M.L., Newberry, R.D., Stappenbeck, T.S., Mack, M., Cella, M., and Colonna, M. (2015). Unique and redundant functions of NKp46+ ILC3s in models of intestinal inflammation. J. Exp. Med. *212*, 1869–1882.

Sonnenberg, G.F., Monticelli, L.A., Elloso, M.M., Fouser, L.A., and Artis, D. (2011). CD4(+) lymphoid tissue-inducer cells promote innate immunity in the gut. Immunity *34*, 122–134.

Stuart, T., Butler, A., Hoffman, P., Hafemeister, C., Papalexi, E., Mauck, W.M., Hao, Y., Stoeckius, M., Smibert, P., and Satija, R. (2019). Comprehensive Integration of Single-Cell Data. Cell *177*, 1888–1902.

Tamoutounour, S., Henri, S., Lelouard, H., de Bovis, B., de Haar, C., van der Woude, C.J., Woltman, A.M., Reyal, Y., Bonnet, D., Sichien, D., et al. (2012). CD64 distinguishes macrophages from dendritic cells in the gut and reveals the Th1-inducing role of mesenteric lymph node macrophages during colitis. Eur. J. Immunol. *42*, 3150–3166.

Tomasek, J.J., Gabbiani, G., Hinz, B., Chaponnier, C., and Brown, R.A. (2002). Myofibroblasts and mechano-regulation of connective tissue remodelling. Nat. Rev. Mol. Cell Biol. *3*, 349–363.

Traag, V.A., Waltman, L., and van Eck, N.J. (2019). From Louvain to Leiden: guaranteeing well-connected communities. Sci. Rep. *9*, 5233.

Ucero, A.C., Bakiri, L., Roediger, B., Suzuki, M., Jimenez, M., Mandal, P., Braghetta, P., Bonaldo, P., Paz-Ares, L., Fustero-Torre, C., et al. (2019). Fra-2-expressing macrophages promote lung fibrosis in mice. J. Clin. Invest. *129*, 3293–3309.

Valentine, J.F., Fedorak, R.N., Feagan, B., Fredlund, P., Schmitt, R., Ni, P., and Humphries, T.J. (2009). Steroid-sparing properties of sargramostim in patients with corticosteroid-dependent Crohn's disease: a randomised, double-blind, placebo-controlled, phase 2 study. Gut *58*, 1354–1362.

VanDussen, K.L., Carulli, A.J., Keeley, T.M., Patel, S.R., Puthoff, B.J., Magness, S.T., Tran, I.T., Maillard, I., Siebel, C., Kolterud, Å., et al. (2012). Notch signaling modulates proliferation and differentiation of intestinal crypt base columnar stem cells. Development *139*, 488–497.

Vanhove, W., Peeters, P.M., Staelens, D., Schraenen, A., Van der Goten, J., Cleynen, I., De Schepper, S., Van Lommel, L., Reynaert, N.L., Schuit, F., et al. (2015). Strong upregulation of AIM2 and IFI16 inflammasomes in the mucosa of patients with active inflammatory bowel disease. Inflamm. Bowel Dis. *21*, 2673–2682.

Weitkamp, B., Cullen, P., Plenz, G., Robenek, H., and Rauterberg, J. (1999). Human macrophages synthesize type VIII collagen in vitro and in the atherosclerotic plaque. FASEB J. *13*, 1445–1457.

Wolf, F.A., Angerer, P., and Theis, F.J. (2018). SCANPY: large-scale single-cell gene expression data analysis. Genome Biol. *19*, 15.

Wolock, S.L., Lopez, R., and Klein, A.M. (2019). Scrublet: Computational Identification of Cell Doublets in Single-Cell Transcriptomic Data. Cell Syst. *8*, 281–291.

Wynn, T.A., and Vannella, K.M. (2016). Macrophages in Tissue Repair, Regeneration, and Fibrosis. Immunity *44*, 450–462.

Xue, J., Schmidt, S.V., Sander, J., Draffehn, A., Krebs, W., Quester, I., De Nardo, D., Gohel, T.D., Emde, M., Schmidleithner, L., et al. (2014). Transcriptome-based network analysis reveals a spectrum model of human macrophage activation. Immunity *40*, 274–288.

Young, M.D., Mitchell, T.J., Vieira Braga, F.A., Tran, M.G.B., Stewart, B.J., Ferdinand, J.R., Collord, G., Botting, R.A., Popescu, D.M., Loudon, K.W., et al. (2018). Single-cell transcriptomes from human kidneys reveal the cellular identity of renal tumors. Science *361*, 594–599.

Zigmond, E., Varol, C., Farache, J., Elmaliah, E., Satpathy, A.T., Friedlander, G., Mack, M., Shpigel, N., Boneca, I.G., Murphy, K.M., et al. (2012). Ly6C hi monocytes in the inflamed colon give rise to proinflammatory effector cells and migratory antigen-presenting cells. Immunity *37*, 1076–1090.

Zigmond, E., Bernshtein, B., Friedlander, G., Walker, C.R., Yona, S., Kim, K.W., Brenner, O., Krauthgamer, R., Varol, C., Müller, W., and Jung, S. (2014). Macrophage-restricted interleukin-10 receptor deficiency, but not IL-10 deficiency, causes severe spontaneous colitis. Immunity *40*, 720–733.

STAR★METHODS

KEY RESOURCES TABLE

REAGENT or RESOURCE	SOURCE	IDENTIFIER
Antibodies		
Anti-mouse B220 antibody (RA3-6B2)	Thermo Fisher Scientific	Cat#25-0452-82; RRID:AB_469627
Anti-mouse CD3 antibody (145-2C11)	Thermo Fisher Scientific	Cat#11-0031-82; RRID:AB_464882
Anti-mouse CD4 antibody (GK1.5)	Thermo Fisher Scientific	Cat#47-0041-82; RRID:AB_11218896
Anti-mouse CD11b antibody (M1/70)	Thermo Fisher Scientific	Cat#11-0112-82; RRID:AB_464935
Anti-mouse CD11c antibody (N418)	Thermo Fisher Scientific	Cat#25-0114-82; RRID:AB_469590
Anti-mouse CD19 antibody (6D5)	Biolegend	Cat#115543; RRID:AB_11218994
Anti-mouse CD45.1 antibody (A20)	Thermo Fisher Scientific	Cat#A15415; RRID:AB_2534428
Anti-mouse CD45.2 antibody (104)	Thermo Fisher Scientific	Cat#A14736; RRID:AB_2534252
Anti-mouse CD90.2 antibody (30-H12)	Thermo Fisher Scientific	Cat#46-0903-82; RRID:AB_10670882
Anti-mouse CD117 antibody (ACK2)	Thermo Fisher Scientific	Cat#12-1172-81; RRID_AB_465815
Anti-mouse CD127 antibody (A7R34)	Biolegend	Cat#135014; RRID:AB_1937265
Anti-mouse CX3CR1 antibody (SA011F11)	Biolegend	Cat#149005; RRID:AB_2564314
Anti-mouse F4/80 antibody (BM8)	Thermo Fisher Scientific	Cat#11-4801-82; RRID:AB_2637191
Anti-mouse GATA3 antibody (TWAJ)	Thermo Fisher Scientific	Cat#50-8899-42; RRID:AB_10596663
Anti-mouse GM-CSF antibody (MP1-22E9)	BD biosciences	Cat#554406; RRID:AB_395371
Anti-mouse IL-17A antibody (TC11-18H10.1)	Biolegend	Cat#506903; RRID:AB_315463
Anti-mouse IL-22 antibody (IL22JOP)	Thermo Fisher Scientific	Cat#17-7222-82; RRID:AB_10597583
Anti-mouse Ly6C antibody (HK1.4)	Thermo Fisher Scientific	Cat#45-5932-82; RRID:AB_2723343
Anti-mouse Ly6C/G antibody (RB6-8C5)	Thermo Fisher Scientific	Cat#47-5931-82; RRID:AB_1518804
Anti-mouse MHC-II (I-A/I-E) antibody (M5/114.15.2)	Thermo Fisher Scientific	Cat#62-5321-82; RRID:AB_2688070
Anti-mouse pro-IL-1β antibody (NJTEN3)	Thermo Fisher Scientific	Cat#17-7114-80; RRID:AB_10670739
Anti-mouse RORγt antibody (Q31-378)	BD biosciences	Cat#564723; RRID:AB_2738916
Anti-mouse TCR beta antibody (H57-597)	Thermo Fisher Scientific	Cat#48-5961-82; RRID:AB_11039532
Anti-mouse TCR gamma/delta antibody (GL3)	Biolegend	Cat#118101; RRID:AB_313826
InVivoMab anti-mouse CD90.2 antibody (30H12)	BioXCell	Cat#BE0066; RRID:AB_1107682
InVivoMab anti-mouse GM-CSF antibody (MP1-22E9)	BioXCell	Cat#BE0259; RRID:AB_2687738
InVivoMab anti-keyhole limpet hemocyanin antibody (LTF-2)	BioXCell	Cat#BE0090; RRID:AB_1107780
Bacterial and Virus Strains		
Citrobacter rodentium ICC180	Dr. S Clare	N/A
Chemicals, Peptides, and Recombinant Proteins		
Recombinant mouse GM-CSF	Peprotech	Cat#315-03
Recombinant mouse M-CSF	Peprotech	Cat#315-02
Recombinant human M-CSF	Peprotech	Cat#300-25
Brefeldin A	Thermo Fisher Scientific	Cat#00-4506-51
LIVE/DEAD Fixable Aqua Dead Cell Stain	Thermo Fisher Scientific	Cat#L34957
Dextran sodium sulfate	MP Biomedicals	Cat#0216011080
Percoll GE Healthcare	Sigma-Aldrich	Cat#17-0891-01
Collagenase A	Sigma-Aldrich	Cat#10103578001

(*Continued on next page*)

Continued

REAGENT or RESOURCE	SOURCE	IDENTIFIER
DNase I from bovine pancreas	Roche	Cat#10104159001
Wheat Germ Agglutinin, Alexa Fluor 594 conjugate	Thermo Fisher Scientific	Cat#W11262
2-deoxy-D-glucose	Sigma-Aldrich	Cat#D6134
PureCol EZ Gel	Sigma-Aldrich	Cat#5074
Critical Commercial Assays		
Intracellular Fixation and Permeabilization Buffer Set	Thermo Fisher Scientific	Cat#88-8824-00
FoxP3 staining buffer set	Thermo Fisher Scientific	Cat#00-5523-00
High Capacity RNA-to-cDNA kit	Applied Biosystems	Cat#4387406
Mouse GM-CSF ELISA kit	R&D systems	Cat#DY415-05
Mouse IL-1β ELISA kit	R&D systems	Cat#DY401-05
Mouse IL-22 ELISA kit	R&D systems	Cat#DY582-05
Mouse PDGF-BB ELISA kit	R&D systems	Cat#DY8464-05
PureLink RNA Mini kit	Thermo Fisher Scientific	Cat#12183025
RNeasy Micro kit	QIAGEN	Cat#74004
ROX Low KAPPA Library Quantification kit	KAPPA Biosystems	Cat#KK4873
SMARTer stranded total RNA-Seq mammalian pico input kit	Takara	Cat#635007
TaqMan Fast Advanced Master Mix	Thermo Fisher Scientific	Cat#4444557
TaqMan Gene Expression (18S rRNA)	Thermo Fisher Scientific	Mm03928990_g1
TaqMan Gene Expression (*Bhlhe40*)	Thermo Fisher Scientific	Mm00478593_m1
TaqMan Gene Expression (*Col4a1*)	Thermo Fisher Scientific	Mm01210125_m1
TaqMan Gene Expression (*Gapdh*)	Thermo Fisher Scientific	Mm99999915_g1
TaqMan Gene Expression (*H2-Aa*)	Thermo Fisher Scientific	Mm00439211_m1
TaqMan Gene Expression (*Hk2*)	Thermo Fisher Scientific	Mm00443385_m1
TaqMan Gene Expression (*Hprt*)	Thermo Fisher Scientific	Mm03024075_m1
TaqMan Gene Expression (*Il1b*)	Thermo Fisher Scientific	Mm00434228_m1
TaqMan Gene Expression (*Il6*)	Thermo Fisher Scientific	Mm00446190_m1
TaqMan Gene Expression (*Il10*)	Thermo Fisher Scientific	Mm01288386_m1
TaqMan Gene Expression (*Il18*)	Thermo Fisher Scientific	Mm00434226_m1
TaqMan Gene Expression (*Il23a*)	Thermo Fisher Scientific	Mm00518984_m1
TaqMan Gene Expression (*Nos2*)	Thermo Fisher Scientific	Mm00518984_m1
TaqMan Gene Expression (*Pdgfb*)	Thermo Fisher Scientific	Mm00440677_m1
TaqMan Gene Expression (*Reg3b*)	Thermo Fisher Scientific	Mm00440616_g1
TaqMan Gene Expression (*Reg3g*)	Thermo Fisher Scientific	Mm00441127_m1
TaqMan Gene Expression (*Slc2a1*)	Thermo Fisher Scientific	Mm00441480_m1
TaqMan Gene Expression (*Tgfbr2*)	Thermo Fisher Scientific	Mm03024091_m1
TaqMan Gene Expression (*Tnfsf15*)	Thermo Fisher Scientific	Mm00770031_m1
Deposited Data		
RNaseq data (colonic macrophages ± anti-CD90.2)	This paper	GEO: GSE135492
RNaseq data (colonic tissue ± anti-CD90.2)	This paper	GEO: GSE135792
RNaseq data (DSS macrophages)	Castro-Dopico et al., 2019	GEO: GSE109040
RNaseq data (human ileal CD biopsies)	Marigorta et al., 2017	GEO: GSE93624
Single cell RNaseq (ileal Crohn's disease)	Martin et al., 2019	GEO: GSE134809
Single cell RNaseq (ulcerative colitis)	Smillie et al., 2019	https://singlecell.broadinstitute.org/single_cell/study/SCP259
Single cell RNaseq (healthy colon)	James et al., 2020	https://www.gutcellatlas.org/

(*Continued on next page*)

Continued

REAGENT or RESOURCE	SOURCE	IDENTIFIER
Single cell RNaseq (murine DSS colon)	Kinchen et al., 2018	GEO: GSE114374
Microarray data (human UC biopsies)	Vanhove et al., 2015	GEO: GSE59071
Microarray data (GM-CSF-treated BMDM)	Lacey et al., 2012	ArrayExpress: E-MTAB-792
Microarray data (Immgen Consortium Phase 2)	Various	GEO: GSE37448
Microarray data (human macrophage stimulations)	Xue et al., 2014	GEO: GSE47189
Experimental Models: Cell Lines		
Mouse: Immortalized lung fibroblasts	John O'Neill	N/A
Experimental Models: Organisms/Strains		
Mouse: C57BL/6 (B6)	Jackson Laboratories	Stock No: 000664
Mouse: B6-GFP: C57BL/6-Tg(UBC-GFP) 30Scha/J	Jackson Laboratories	Stock No: 004353
Mouse: *Csf2rb*$^{-/-}$: B6.*Csf2rb*$^{-/-}$	Fiona Powrie	N/A
Mouse: CD45.1^{+}: B6.SJL-*Ptprc*^{a} *Pepc*^{b}/BoyJ	Jackson Laboratories	Stock No: 002014
Mouse: *Rag2*$^{-/-}$: B6(Cg)-*Rag2*$^{tm1.Cgn}$/J	Jackson Laboratories	Stock No: 008449
Mouse: *Rag1*$^{-/-}$: B6.129S7-*Rag1*^{tm1Mom}/J	Jackson Laboratories	Stock No: 002216
Mouse: RORγt-EGFP: B6.RORγt-EGFP	Andrew McKenzie	N/A
Software and Algorithms		
FlowJo	Tree Star Inc.	https://www.flowjo.com/
Gene Set Enrichment Analysis	Broad Institute	https://www.gsea-msigdb.org/gsea/
GraphPad Prism 6	GraphPad Software	https://www.graphpad.com/
ImageJ	National Institutes of Health	https://imagej.nih.gov/ij/
Ingenuity Pathway Analysis	QIAGEN	http://www.qiagen.com/cn/
Other		
123count eBeads	Thermo Fisher Scientific	Cat#01-1234-42

RESOURCE AVAILABILITY

Lead Contact

Further information and requests for resources and reagents should be directed to and will be fulfilled by the Lead Contact, Menna R. Clatworthy (mrc38@cam.ac.uk).

Materials Availability

This study did not generate new unique reagents.

Data and Code Availability

RNA sequencing datasets generated in this study have been deposited on Gene Expression Omnibus under accession codes GSE135792 and GSE135492.

EXPERIMENTAL MODEL AND SUBJECT DETAILS

All mouse lines used here are on a C57BL/6J background. CD45.2^{+}, CD45.1^{+}, and *Rag2*$^{-/-}$ and *Rag1*$^{-/-}$ mice, and mice expressing EGFP under the control the of ubiquitin C promoter, were obtained from Jackson Laboratories (Margate, UK) and maintained inhouse for several generations. RORγt-EGFP mice were kindly provided by Andrew McKenzie (MRC-LMB, UK) and maintained in house for several generations. *Csf2rb*$^{-/-}$ mice were kindly provided by Fiona Powrie (University of Oxford, UK). For the generation of bone marrow chimeras, recipient CD45.1/2^{+} C57BL/6 mice were lethally irradiated (2 × 5.5 G) followed by immediate tail intravenous (i.v.) injection of 2 × 10^{6} bone marrow cells from CD45.2^{+} *Csf2rb*$^{-/-}$ (all chimeras), CD45.1^{+} *Csf2rb*$^{+/+}$ (80:20 chimeras), or CD45.2^{+} *Csf2rb*$^{+/+}$ (100% chimeras) mice. Recipient mice were checked for reconstitution after 8 weeks by tail vein bleed and flow cytometry on peripheral leukocytes prior to commencement of colitis experiments. For all *in vivo* colitis experiments, littermate mice were sex-matched within experiment and co-housed throughout. Both male and female 8-14 week old mice were used. Mice

were maintained in specific pathogen-free conditions at a Home Office-approved facility in the UK, at the University of Cambridge or the Wellcome Trust Sanger Institute. All procedures were carried out in accordance with the United Kingdom Animals (Scientific Procedures) Act of 1986.

METHOD DETAILS

DSS-Induced Experimental Colitis

Colitis was induced the addition of 2% (*w/v*) 36,000-50,000 MW DSS (MP Biomedicals) to drinking water for 6-7 days, unless otherwise stated. Depleting/blocking antibodies were administered *in vivo* via i.p. injection (final volume, 200 mL sterile PBS). 0.25 mg InVivoMab anti-CD90.2 IgG (30H12; BioXCell) was given on days 0 and 3 of DSS protocol. 0.5 mg anti-GM-CSF IgG antibody (MP1-22E9; BioXCell) was injected on days −3, 0 and 3 of DSS protocol. InVivoMAb anti-KHL IgG (LTF-2; 2BScientific) was used as a control. Colitis severity was monitored daily through changes in body weight, stool consistency, and intestinal hemorrhage. Moderate severity limits were imposed, with 20% weight loss or two moribund characteristics judged to be the severity threshold. At experimental endpoints, colon, spleen, MLN and blood were harvested and colitis severity further assessed through morphological changes in organs. The spleen, MLN, and colon were weighed, and colon length measured from cecum to rectum to determine length. The tissues were then processed for histology, RNA extraction, or flow cytometric analysis.

Citrobacter rodentium Infection

Mice were orally infected with 200 μL of *Citrobacter rodentium* ICC180 (10^9 CFUs). At day 7-10, mice were sacrificed, and colon processed for downstream applications. For 100% chimera experiments, *C. rodentium* infection was prolonged until disease severity limits (20% weight loss) were achieved. Antibody dosing was carried out as described above. Caecum and liver were homogenized, and bacterial burden assessed by serial dilution, plating onto LB agar plates, and overnight incubation at 37°C.

Murine Primary Cell Isolation

Spleen, mesenteric lymph nodes (MLN), and colon were harvested and processed for single cell suspensions. Colons were dissociated from fat and luminal contents were gently removed. Tissues were opened longitudinally, cut into 0.5 cm pieces and washed by vortexing in ice-cold PBS with 10 mM HEPES. Tissue pieces were subsequently incubated with a stripping solution (RPMI-1640 medium containing 2% (*v/v*) FCS, 10 mM HEPES, 1 mM DTT, and 5 mM EDTA) at 37°C for two intervals of 20 min to remove epithelial cells, prior to enzymatic digestion in RPMI-1640 medium containing 1 mg/ml collagenase A (Sigma Aldrich) and 60 mg/mL DNase I (Roche). Tissue suspensions were mechanically dissociated and passed through a 70 mm cell strainer. Intestinal single cell suspensions were then harvested at the interface of a 40/80% (*v/v*) Percoll (Sigma-Aldrich) gradient and washed thoroughly in ice-cold PBS containing 3% (*v/v*) FCS before proceeding to further analysis. MLN and spleen suspensions were harvested by enzymatic digestion and mechanical tissue dissociation through a 70 mm filter. Splenic suspensions were subjected to red blood cell lysis (distilled H_2O containing 0.83% (*w/v*) NH_4Cl, 0.1% (*w/v*) $NaHCO_3$, 100 mM EDTA) prior to washing twice in ice-cold PBS for analysis.

Peripheral blood leukocytes were obtained by tail vein bleed into Microvette EDTA-coated tubes (Sarstedt). Red blood cells were lysed as above prior to washing twice in ice-cold PBS for subsequent downstream analysis.

Flow Cytometry and Cell Sorting

Single cell suspensions were blocked for 20 min at 4°C with 0.5% (*v/v*) heat-inactivated mouse serum followed by extracellular staining for 30 min at 4°C with a combination of the following antibodies in 96-well plates. Murine antibodies: B220 (RA3-6B2, Thermo Fisher Scientific), CD3ε (145-2C11, Thermo Fisher Scientific), CD4 (GK1.5, Thermo Fisher Scientific), CD11b (M1/70, Thermo Fisher Scientific), CD11c (N418, Thermo Fisher Scientific), CD19 (6D5, Biolegend), CD45.1 (A20, Thermo Fisher Scientific), CD45.2 (104, Thermo Fisher Scientific), CD90.2 (30-H12, Thermo Fisher Scientific), CD117 (ACK2, Thermo Fisher Scientific), CD127 (A7R34, Biolegend), CX3CR1 (SA011F11, Biolegend), F4/80 (BM8, Thermo Fisher Scientific), Ly6C (HK1.4, Thermo Fisher Scientific), Ly6C/G (RB6-8C5, Thermo Fisher Scientific), MHC-II (M5/114.15.2, Thermo Fisher Scientific), TCR beta (H57-597, Thermo Fisher Scientific), and TCR gamma/delta (GL3, Biolegend). Antibodies were used at a dilution of 1:100-200 in PBS. Viability staining was performed with LIVE/DEAD Fixable Aqua Dead Cell Stain kit (Thermo Fisher Scientific) for 20 min at room temperature. For intracellular cytokine staining, cells were incubated in RPMI-1640 medium containing 10% FCS, 1X penicillin-streptomycin (both Sigma-Aldrich), and 1X Brefeldin A (Thermo Fisher Scientific) solution for 4 h at 37°C, prior to fixation and permeabilization using the Intracellular Fixation and Permeabilization Buffer Set (Thermo Fisher Scientific) as per the manufacturer's instructions. For chimera IL-17A and IL-22 staining, cells were additionally treated with 20 ng/ml PMA (Sigma Aldrich) and 1 μg/ml ionomycin (Sigma Aldrich) for 4 h at 37°C. Staining was carried out for 1 h at room temperature using a combination of the following antibodies: GM-CSF (MP1-22E9, BD biosciences), IL-17A (TC11- 18H10.1, Biolegend), IL-22 (IL22JOP, Thermo Fisher Scientific), and pro-IL-1β (NJTEN3, Thermo Fisher Scientific). All antibodies were used at a 1:100 dilution. For intracellular transcription factor staining, the FoxP3 staining buffer set (Thermo Fisher Scientific) was use according to the manufacturer's instructions. Staining was performed with the following antibodies for 1 h at room temperature: RORγt (Q31-378, BD biosciences) and GATA3 (TWAJ, Thermo Fisher Scientific). All antibodies were used at a 1:100 dilution. Cell counting was performed using 123count eBeads (Thermo Fisher Scientific). Flow cytometry data collection was performed on a Fortessa cytometer (BD biosciences) and data was analyzed using FlowJo software (Tree Star Inc.).

Murine intestinal macrophages were flow-sorted as live CD11b$^+$ CX3CR1$^+$ Ly6C^{lo} MHC-II^{hi} cells. Murine intestinal ILC3s were sorted as live CD45.2^{int} lineage (CD3, CD19, B220, CD11b, Gr-1, CD11c, TCRb, TCRgd)$^-$ RORγt$^+$ (using RORγt-GFP reporter mice) or live CD45.2^{int} lineage$^-$ CD117$^+$ CD127$^+$ (using wild-type mice). Cell sorting was performed on iCyt Synergy (Sony Biotechnology Inc.), and MoFlo (Beckman Coulter) cell sorters. Data were analyzed using FlowJo software (Tree Star Inc.).

Macrophage Cultures and Stimulation

For murine BMDMs, bone marrow was flushed from the femur and tibia of mice using ice-cold sterile PBS and the subsequent cell suspension treated with red cell lysis buffer. Treated cells were then washed in ice-cold sterile PBS. BMDMs were generated by incubation of bone marrow cells in RPMI-1640 medium containing 10% FCS and 1X penicillin-streptomycin (both Sigma-Aldrich) (referred to as complete RPMI (cRPMI)) supplemented with 100 ng/ml murine macrophage colony-stimulating factor (M-CSF; Peprotech). M-CSF-supplemented culture medium was replaced on day 3 and adherent BMDMs were harvested on day 5-6.

For cytokine stimulation experiments, plated BMDMs were stimulated with 20 ng/ml murine GM-CSF (Peprotech) or control media (cRPMI supplemented with 20 ng/ml M-CSF) for 16 h. For macrophage-ILC3 co-cultures, 2.5-5 × 10^4 BMDMs were plates in flat-bottomed 96-well plates (Sigma-Aldrich) in cRPMI supplemented with 20 ng/ml M-CSF overnight before co-culture with flow-sorted intestinal ILC3s (2:1 ratio of macrophages:ILC3s) or ILC3-conditoned media. After 24-72 h, cells were harvested or used for downstream assays. ILC3-conditioned media was generated by culture of ILC3s in cRPMI supplemented with 50 ng/ml murine IL-1β (Peprotech) for 16 h at a concentration of 1 × 10^6 ILC3s/ml cRPMI. cRPMI supplemented with IL-1β was used as a control in these experiments.

Commensal Microbe Phagocytosis

For commensal labeling, faecal content was extruded from the colon of WT mice, mechanically homogenized in PBS and vortexed for 5 min to break up clumps. Large remaining pieces were spun down in a desktop centrifuge for 20 s at 3,000 rpm and supernatant passed through a 30 μm cell filter to obtain a crude commensal bacteria extract. Commensal bacteria were then washed in PBS three times by spinning in a large centrifuge at 4,200 rpm for 10 min. Bacteria were fixed with 2.5% PFA at RT for 10 min, spun down and resuspended in 0.1% Triton X-100 in PBS for 10 mins at RT. Bacteria were labeled by incubating with 100 μg/ml Wheat Germ Agglutinin, Alexa Fluor 594 conjugate (W11262, Thermo Fisher Scientific) for 6 h at RT, washed thoroughly, and post-fixed for 10 min in 2.5% PFA at RT, and resuspended in 1 mL PBS before use in phagocytosis assays. For phagocytosis assays, BMDMs or colonic lamina propria cells were incubated with 1/100 dilution of the fluorescently labeled commensal bacterial extract for 30 min. Cells were then washed 3 times in PBS before downstream flow cytometry analysis.

Macrophage Commensal Stimulation

2.5 × 10^5 BMDMs were seeded into 24-well plates and stimulated with GM-CSF or control media as indicated above for 24 h. Murine faecal commensal suspensions were prepared from a 100 mg/ml stool sample in PBS and processed as above. BMDMs were then stimulated with 1:100 dilution of crude commensal suspension for 6 h and supernatants collected for cytokine analysis. For 2-deoxy-D-glucose assays (2DG), BMDMs were pre-treated for 1 h with 1 mM 2DG (Sigma Aldrich) prior to commensal stimulation.

Macrophage Motility Assay

For analyzing BMDM seek behavior, 0.5 × 10^6 BMDMs stimulated for 24 hr with 20 ng/ml GM-CSF or 50 ng/ml M-CSF from EGFP-ubiquitin mice were re-suspended in 100 μL of cRPMI and mixed with 200 μL PureCol EZ Gel (5074, Sigma-Aldrich). The collagen cell mix was then applied to a three-sided chamber on a microscopy slide, created by sealing with paraffin wax and covering with a glass slip. The microscopy slide was then incubated upright in a 5% CO_2, 37°C incubator for 1 h to allow the collagen gel to set. 250 μL of cRPMI was then layered on top of the collagen gel inside the chamber, the remaining side sealed with paraffin wax and slide taken immediately to a TCS SP8 (Leica) microscope containing a 37°C pre-heated imaging chamber for imaging of BMDM seek behavior over the space of 1 h. Seek behavior videos were processed and quantified using Imaris software.

Fibroblast Wound Scratch Assay

Murine immortalized lung fibroblasts, a gift from John O'Neill (MRC-LMB, Cambridge, UK) were seeded at 4 × 10^4 cells per well into 96-well flat-bottomed plates overnight and subsequently scratched once with a p200 pipette tip. Scratched fibroblasts were washed in DMEM and subsequently incubated with macrophage-conditioned DMEM medium or DMEM supplemented with 20 ng/ml recombinant murine PDGF-BB (Peprotech). Scratched fibroblasts were imaged at 4 h intervals for 24 h on an IncuCyte S3 (Essen Bioscience) to monitor wound closure. Macrophage-conditioned medium was generated by incubation of control or GM-CSF-primed BMDMs in fresh DMEM medium for 4 h. Image analysis was performed in ImageJ.

Metabolic Assays

7 × 10^4 BMDMs per well were seeded into standard Seahorse 96-well plates and cultured with ILC3-conditioned cRPMI or control cRPMI ± anti-GM-CSF IgG (BioXCell) for 16 h. Media was changed prior to analysis for glucose-free XF base media (Seahorse Biosciences) supplemented with 2 mM sodium pyruvate (Thermo Fisher Scientific) and 1X glutamax (Thermo Fisher Scientific).

Perturbation of metabolic pathways was achieved by addition of glucose (10 mM), oligomycin (1 μM), and 2-deoxy-D-glucose (100 mM) (Sigma-Aldrich) and data collected on a Seahorse XFp Analyzer (Agilent Technologies).

RNA Extraction and Reverse Transcription

RNA extraction was carried out using commercially available kits as per the manufacturer's instructions. QIAGEN RNeasy micro kits were used for cell numbers below 5×10^5. The PureLink RNA mini kit (Thermo Fisher Scientific) was used for cell numbers over 5×10^5. For whole tissue RNA extraction, tissue pieces were first disrupted using a Precellys 24 Homogenizer (Bertin Instruments), before extraction using the PureLink RNA mini kit (Thermo Fisher Scientific). RNA concentration and purity were determined using a Nano-Drop spectrophotometer (Thermo Scientific) prior to cDNA synthesis using a High-Capacity RNA-to-cDNA kit (Applied Biosystems).

Quantitative Polymerase Chain Reaction

All qPCR was carried out in triplicate in a final volume of 10 μL in a 384-well plate with Taqman reagents and the following pre-designed TaqMan Gene Expression Assay primers and probes (Thermo Fisher Scientific). Murine primers: 18S rRNA (Mm03928990_g1), *Bhlhe40* (Mm00478593_m1), *Col4a1* (Mm01210125_m1), *Gapdh* (Mm99999915_g1), *H2-Aa* (Mm00439211_m1), *Hk2* (Mm00443385_m1), *Hprt* (Mm03024075_m1), *Il1b* (Mm00434228_m1), *Il6* (Mm00446190_m1), *Il10* (Mm01288386_m1), *Il18* (Mm00434226_m1), *Il23a* (Mm00518984_m1), *Nos2* (Mm00440502_m1), *Pdgfb* (Mm00440677_m1), *Reg3b* (Mm00440616_g1), *Reg3g* (Mm00441127_m1), *Slc2a1* (Mm00441480_m1), *Tgfbr2* (Mm03024091_m1), and *Tnfsf15* (Mm00770031_m1). qPCR was carried performed on the Viia 7 PCR machine (Life Technologies). Gene expression was normalized to *Gapdh*, *Hprt*, or 18S rRNA using the $2^{-\Delta Ct}$. The $2^{-\Delta\Delta Ct}$ method was used for normalization between experimental conditions and genotypes.

ELISA

Quantification of murine cytokines and PDGF-BB in culture supernatants was carried out using commercially available R&D systems Duoset ELISA kits, as per the manufacturer's instructions. Optical densities were measured at 450 nm and 530 nm background using a CLARIOstar spectrophotometer (BMG Labtech).

RNaseq Sample Preparation

Macrophage RNASeq: Flow-sorted colonic CX3CR1$^+$ CD11b$^+$ Ly6C^{lo} MHC-II$^+$ macrophages were immediately lysed in 750 μL RLT plus buffer (QIAGEN). Samples were vortexed, snap frozen on dry ice and stored at −80°C. RNA was extracted from cell lysates using the RNeasy plus micro kit (QIAGEN) as per the manufacturer's instructions. Optimal DNA depletion columns (QIAGEN) were used to remove contaminating genomic DNA. Purified RNA was eluted in nuclease free water (Ambion) and stored at −80°C. Quality and concentration of the purified RNA was assessed using an RNA pico chip (Applied Biosystems) using a Bioanalyzer 2000 (Applied Biosystems) as per the manufacturer's instructions. For all RNaseq experiments, samples had an RNA integrity number > 8. For the preparation of libraries, SMARTer stranded total RNaseq mammalian pico input kit (Takara) was used as per the manufacturer's instructions. To produce the libraries, 5ng of total RNA was used.

Colon RNASeq: RNA was extracted using the PureLink RNA mini kit and contaminating DNA digested with TurboDNase (Invitrogen). Quality of RNA was checked as above. RNASeq librarys were prepared using Illumina TruSeq Stranded total RNA library prep kit wuth 1ug of RNA as per manufactuers instructions.

Library size was assessed with a High Sensitivity DNA chip (Applied Biosystems) using a Bioanalyzer 2000 (Applied Biosystems) as per the manufacturer's instructions. Library concentration was quantified by PCR with ROX low KAPPA library quantification kit (KAPPA Biosystems). Libraries were pooled at an equimolar concentration with up to 10 libraries per pool.

RNA Sequencing and Analysis

Sequencing of the Colon RNASeq was carried out using a Novaseq 6000 (Illumina) and the Macrophage RNaseq on a Hiseq 4000 both on a 2x150bp sequencing run. Sequencing was carried out at Genewiz (NJ, USA). Pooled libraries were de-multiplexed by Genewiz using Casava (Illumina) before transfer of the data to the University of Cambridge. The Fastq files from libraries prepared using the Takara library prep were were trimmed of the first 3 nucleotides of the R1 strand and contaminating adaptor sequences and poor-quality bases removed (bases with a phred 33 score of < 30) using trimgalore! (Babraham bioinformatics). The Illumina library preps were only trimmed for quality. Sequencing quality of the resulting files was assessed using FastQC (Babraham bioinformatics). Fastq files were aligned to the mm10 genome using hisat2. Subsequent analysis was carried out in R. Reads were counted and assigned to genes using the Featurecount function from the RSubread package. Differential expression analysis was carried out using DESeq 2 using a linear model with an appropriate design matrix following the default workflow. Resulting figures were plotted using ggplot2 and pheatmap. GSEA was performed for RNaseq data by first assigning a rank metric to each gene using the following formula:

$$\text{Rank metric} = 1\left(P\text{value} + 1 \times 10^{-300}\right) * \left(|\text{LFC}| \,/\, \text{LFC}\right)$$

GSEA was then run using GSEA 4.0.1 using the pre-ranked option with the classic setting against either gene sets from the molecular signature database or custom 100/200-gene gene sets indicated in the text. STRING analysis was performed using the online portal (https://string-db.org) selecting high-highest confidence parameters (0.700-0.900) with no additional interactors, unless stated in the text.

Public RNaseq and Microarray

Publicly available RNaseq and microarray datasets were downloaded from GEO (https://www.ncbi.nlm.nih.gov/geo/) or ArrayExpress (https://www.ebi.ac.uk/arrayexpress) along with appropriate chip annotation data. All analyses were carried out using R. All datasets were downloaded as raw intensity matrices. For microarray analysis, data was normalized using RMA and limma. Probes were reduced to one probe per gene by selecting the probe with the greatest variance across the samples using the gene filter package. Differential expression was varied out using limma with an appropriate design matrix.

Public bulk transcriptomics datasets used in this study are as follows: GEO: GSE47189 (Human macrophage stimulations) (Xue et al., 2014); GEO: GSE93624 (mucosal biopsies from ileal Crohn's disease) (Marigorta et al., 2017); GEO: GSE59071 (mucosal biopsies from ulcerative colitis) (Vanhove et al., 2015); ArrayExpress: E-MTAB-792 (BMDMs treated with GM-CSF for 24 h) (Lacey et al., 2012); GEO: GSE109040 (colonic murine macrophages from control or DSS-treated mice) (Castro-Dopico et al., 2019); GEO: GSE37448 (Immgen Consortium Phase 2).

Single Cell RNaseq

For ulcerative colitis and healthy colonic scRNaseq analysis, count matrices were downloaded from the Broad Single Cell Portal (https://singlecell.broadinstitute.org/single_cell/study/SCP259) (Smillie et al., 2019) and https://www.gutcellatlas.org/ (James et al., 2020). Data were normalized and log transformed using the Scanpy tool (Wolf et al., 2018). Immune cell data were split by health status or sampling site, and cell type and gene expression values plotted using ggplot2 tools in R.

For ileal CD, single cell RNaseq was extracted from GEO: GSE134809 (Martin et al., 2019). The single-cell data (10x *cellranger* output) was processed using standard *Seurat*-inspired *scanpy* workflow (Stuart et al., 2019; Wolf et al., 2018) with standard quality control steps; cells were filtered if number of genes > 2500 or < 200 and percentage mitochondrial content > = 5%. Genes were retained if is expressed by at least 3 cells. Doublet detection was performed using *scrublet* (Wolock et al., 2019) with adaptations outlined in (Popescu et al., 2019). Batch correction was performed using *bbknn* with samples (patient/donor) as the batch term (Polański et al., 2020). Clustering was performed using Leiden algorithm (Traag et al., 2019). To group clusters into either resident macrophages or infiltrating monocyte/macrophages and DCs, marker genes were selected based on the original paper. Wilcoxon test implemented in *Scanpy (rank_genes_groups)* was performed to extract the list of significant differentially expressed genes between infiltrating and resident macrophages. for IPA analysis.

For murine gut stromal scRNaseq analysis, expression matrices from GSE114374 were acquired from GEO (Kinchen et al., 2018). Highly variable genes were called using the Scanpy workflow in python, setting min_mean = 0.01, max_mean = 8, and min_disp = 1 (Wolf et al., 2018). The expression matrix was subsetted to highly variable genes, before computing dimensionality reduced coordinates using principal components analysis. We used these coordinates to generate a UMAP embedding, and clustered the resulting neighbor graph using Leiden clustering (Traag et al., 2019), setting resolution = 0.5. Consequently, we were able to manually annotate clusters to the cell types identified in Kinchen et al. (2018) on the basis of canonical marker expression as described in their paper. We calculated marker gene sets for each annotated cell type using the tf-idf metric and computed p values using hypergeometric testing (Young et al., 2018).

Ingenuity Pathway Analysis

Ingenuity Pathway Analysis (IPA) was performed using the following settings were used for all samples: Ingenuity knowledge database (Genes only); direct and indirect relationships; 35 molecules per network and 25 networks per analysis; all nodes and datasources; experimentally observed confidence setting; and all species, tissues and cell lines, and mutations. Customised settings for each dataset was used to sample between 500-1000 genes. For DSS-macrophages: minimum expression = 10; fold change = 1.5; adjusted *P value* = 0.05. For αCD90.2 macrophages: minimum expression = 10; fold change = 1.0; adjusted *P value* = 0.05. For ileal Crohn's disease scRNaseq: fold change = 2; adjusted *P value* = 0.05. For upstream regulators, all observations biased observations were excluded and bias-corrected regulation z-score and *P value* of overlap statistics used.

QUANTIFICATION AND STATISTICAL ANALYSIS

Statistical analysis was performed using GraphPad Prism software, R, IPA, or GSEA. For *in vivo* colitis experiments, comparison between experimental groups was performed using a nonparametric Mann-Whitney-U test, unless otherwise stated, and medians are indicated. For *in vitro* stimulation experiments, mean ± standard error of mean (SEM) are shown and a parametric Student's two-tailed t test, and one-way or two-way ANOVA with Tukey's multiple comparisons test was used, unless paired samples were used, where a paired t test was used. For RNaseq bioinformatics analyses, *P value*s were calculated using the standard DESeq 2 method with multiple correction using BH procedure. For microarray experiments, *P value*s were calculated using the limma package with multiple correction using BH procedure. * $p < 0.05$; ** $p < 0.01$; *** $p < 0.001$; **** $p < 0.0001$. Sample sizes (*n*) for all shown data can be found in figure legends. *In vitro* stimulations were performed in triplicate, unless stated, and sample sizes for *in vivo* experiments were determined based on initial experiments.

Cell Reports, Volume *32*

Supplemental Information

GM-CSF Calibrates Macrophage Defense

and Wound Healing Programs

during Intestinal Infection and Inflammation

Tomas Castro-Dopico, Aaron Fleming, Thomas W. Dennison, John R. Ferdinand, Katherine Harcourt, Benjamin J. Stewart, Zaeem Cader, Zewen K. Tuong, Chenzhi Jing, Laurence S.C. Lok, Rebeccah J. Mathews, Anaïs Portet, Arthur Kaser, Simon Clare, and Menna R. Clatworthy

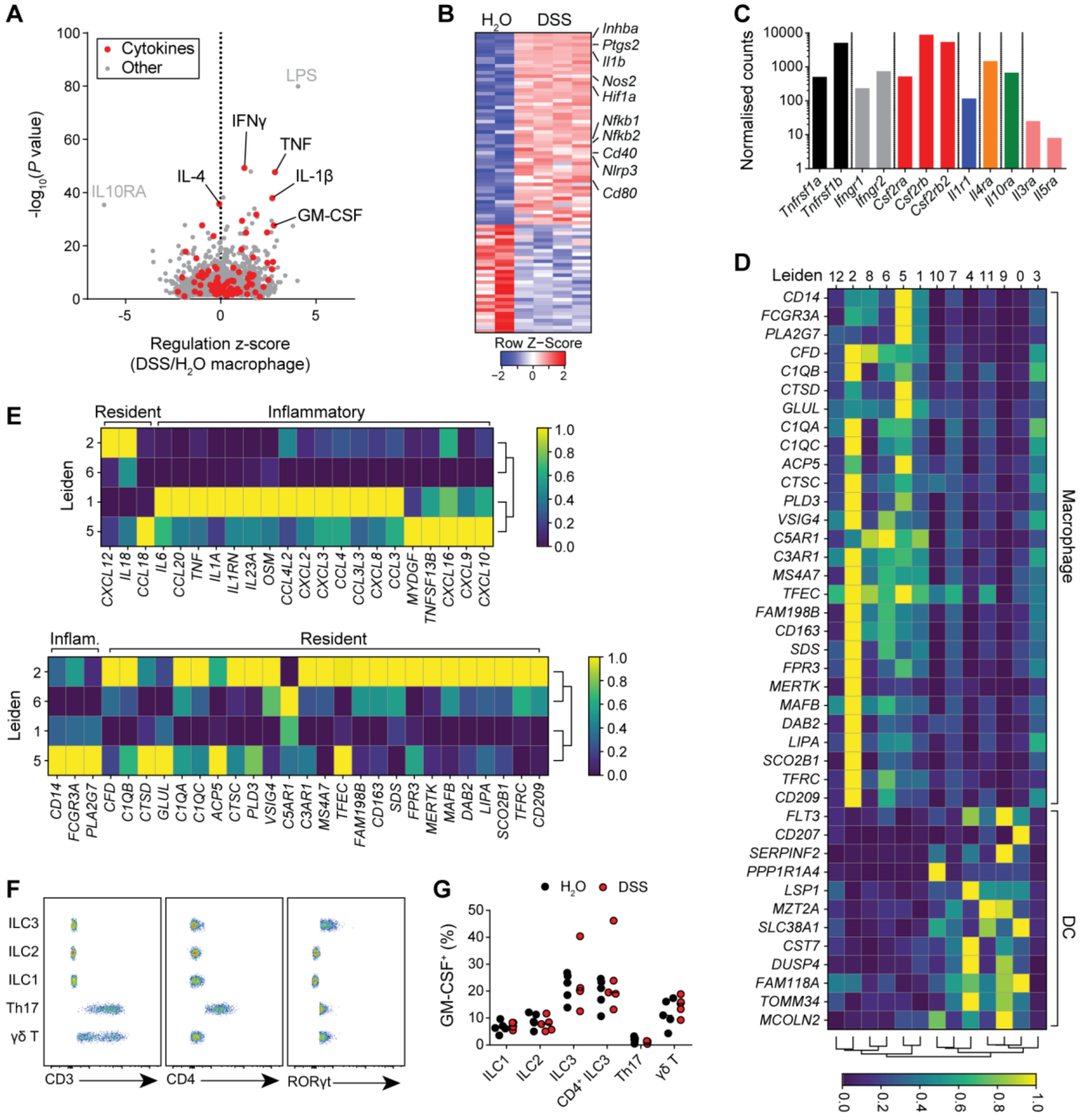

Figure S1. GM-CSF is an upstream regulator of inflammatory macrophage function in the intestine. Related to Figure 1. (**A**) Plot of z-score and *P* value for predicted regulators of DSS vs H_2O macrophage activation, determined by IPA. Data derived from GEO: GSE109040. (**B**) Heatmap of predicted GM-CSF regulated genes in DSS macrophages from dataset in **A**. (**C**) Normalised expression counts of candidate cytokine receptor genes in colonic macrophages from **A**. (**D, E**) Heatmaps of marker genes and cytokines distinguishing (**D**) macrophages from dendritic cells and (**E**) resident from inflammatory macrophages amongst all MNPs in human mucosal biopsies from patients with ileal Crohn's disease, determined by scRNA-seq. Data derived from GEO: GSE134809. (**F**) Surface marker and RORγt expression by colonic ILC and T cell subsets. (**G**) GM-CSF$^+$ cells as a proportion of each immune cell subset from mice treated with H_2O or 2 % DSS for 6 days. *N* = 5 per group. Medians indicated. Data (**F, G**) representative of two independent experiments. *P* values were calculated in IPA software (**A**).

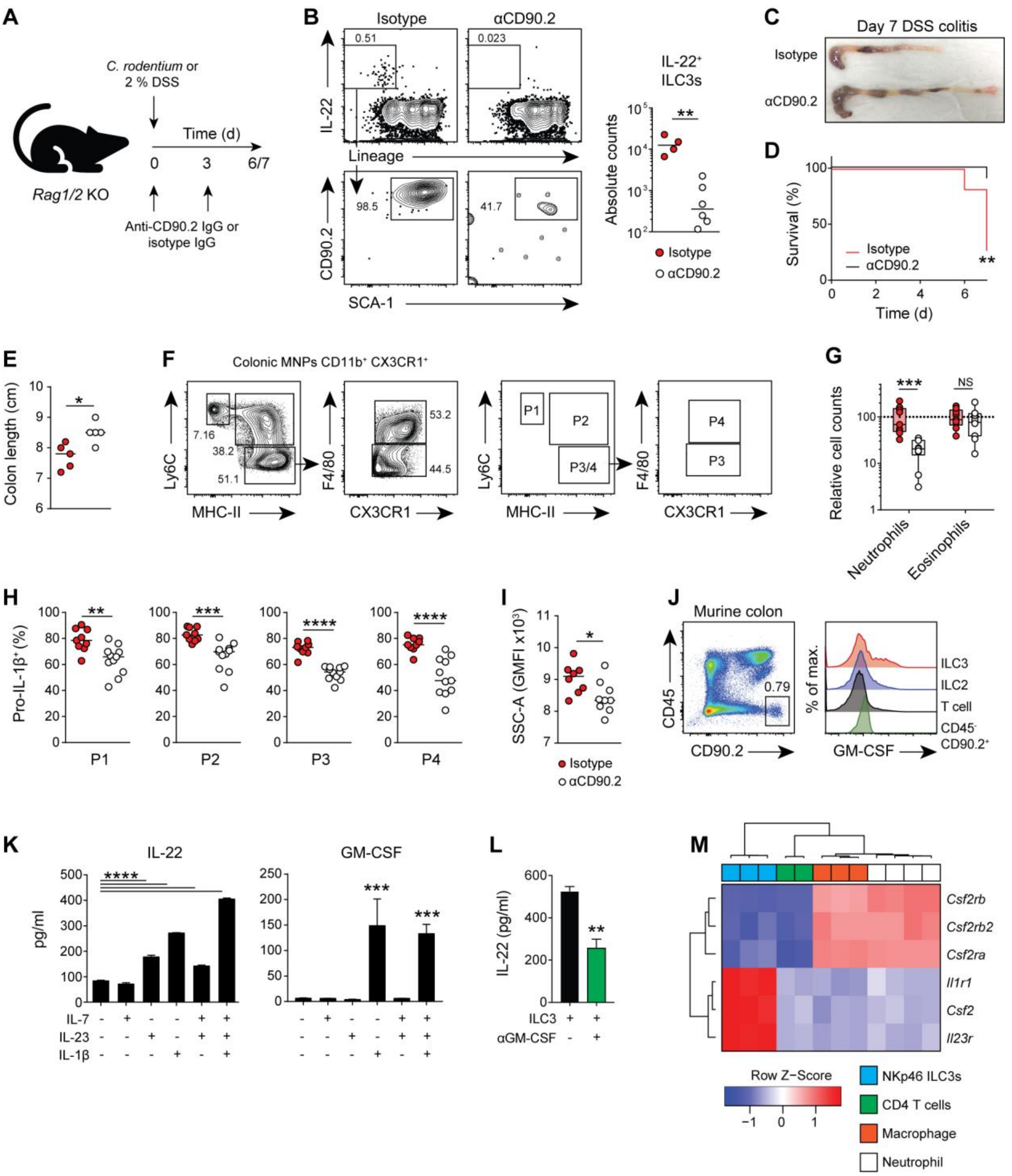

Figure S2. ILCs and innate GM-CSF augment intestinal macrophage activation during enteropathic infection and colitis. Related to Figure 2. (**A**) Schematic of ILC depletion strategy. (**B**) Colonic lineage$^-$ CD90.2$^+$ SCA-1$^+$ IL-22$^+$ ILC3 quantification in 2 % DSS-treated *Rag2*$^{-/-}$ mice, as in **A** (*n* = 4-6 per group). Medians indicated. (**C**) Colon length in *Rag2*$^{-/-}$ mice treated as in **A** at day 7 after 2 % DSS administration. (**D**) Survival of mice treated as in **C** (exceeding 20 % weight loss). *N* = 10-11 per group. (**E**) Colon length in mice treated as in **A**, infected with *C. rodentium* at 7 dpi. *N* = 5 per group. Medians indicated. (**F**) Representative MNP waterfall gating strategy in murine colon. (**G**) Colonic neutrophil and eosinophil relative cell count in *Rag2*$^{-/-}$ mice treated with 2 % DSS ± isotype or anti-CD90.2 IgG for 6 days. *N* = 9-11 per group. Medians indicated. (**H**) Pro-IL-1β expression by colonic MNP subsets (P1-P4) in mice treated as in **B**. Medians indicated. (**I**) SSC of colonic Ly6C^{lo} MHC-II$^+$ MNPs from mice treated as in **A** with *C. rodentium*. Medians indicated. (**J**) GM-CSF expression by non-haematopoietic CD90.2$^+$ CD45.2$^-$ cells in murine colon. Mice treated as in **A**. (**K**) IL-22 and GM-CSF production by flow-sorted intestinal ILC3s cultured with indicated cytokines for 16h. Mean ± SEM indicated. (**L**) IL-22 production in ILC3-BMDM co-cultures ± isotype or anti-GM-CSF IgG after 16h. Mean ± SEM indicated. (**M**) Cytokine and receptor expression by intestinal NKp46$^+$ ILC3s, lymph node CD4$^+$

T cells, lung macrophages and blood neutrophils from the Immgen Consortium Phase 2 (GEO: GSE37448). Data are representative of three independent experiments (**B-I, K, L**), with single (**B, C, E-G, I, K, L**) or two pooled experiments (**D, G, H**) shown. *P* values were calculated using Mann-Whitney U test (**B, E, G-I**), Gehan-Breslow-Wilcoxon test (**D**), a one-way ANOVA with Tukey's multiple comparisons test (**K**), and Student's *t* test (**L**). * $P < 0.05$; ** $P < 0.01$; *** $P < 0.001$; **** $P < 0.0001$.

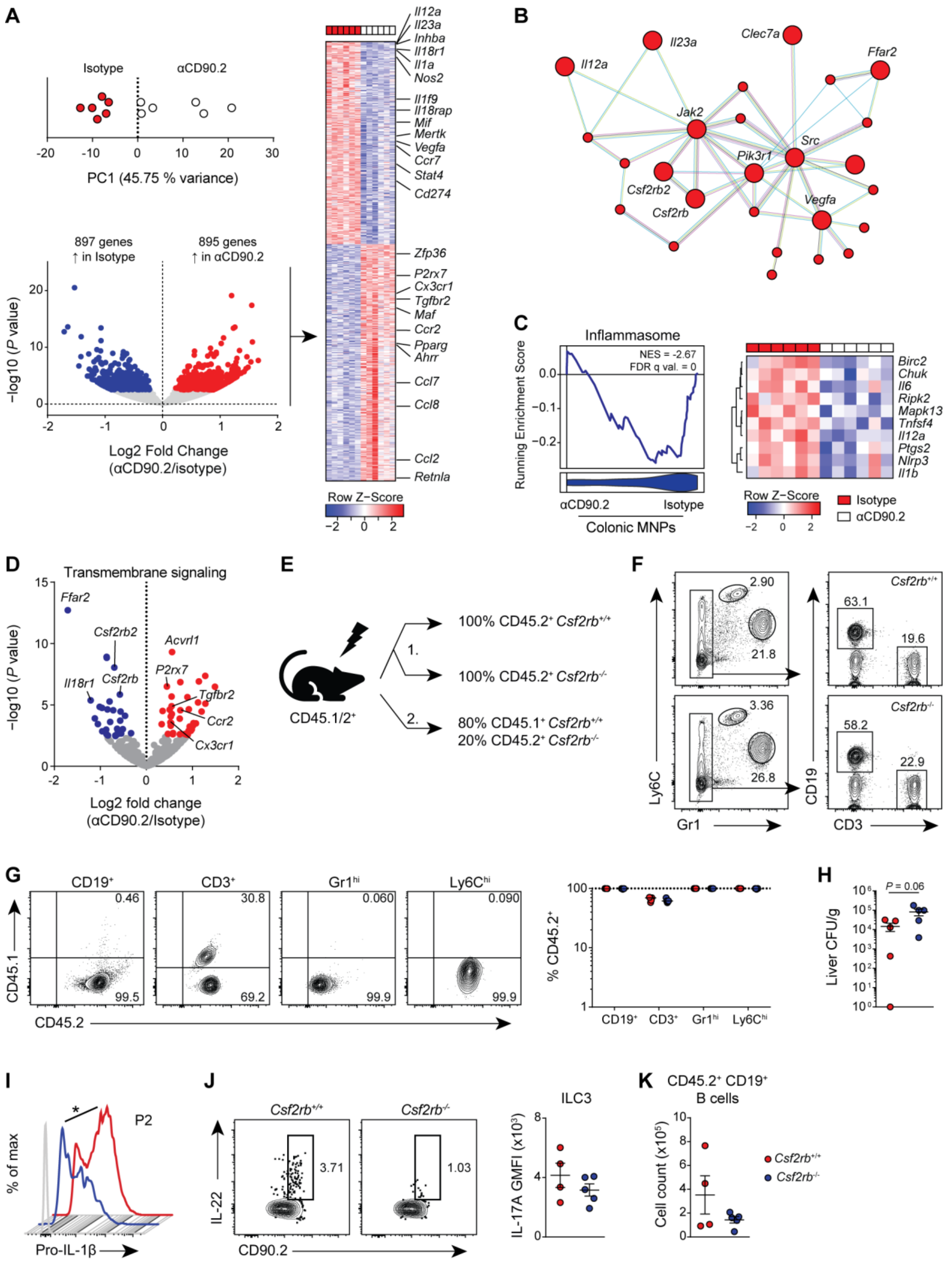

Figure S3. GM-CSF drives inflammatory macrophage function and type 17 immunity *in vivo*. Related to Figure 3. (**A**) Principal component 1 of RNAseq dataset of colonic Ly6C^{lo} MHC-II^{+} macrophages from *Rag2*$^{-/-}$ mice treated with 2 % DSS ± isotype or anti-CD90.2 IgG antibodies (top), and volcano plot and heatmap of all DEGs (adj. *P* val. < 0.05). Selected genes of interest are highlighted. $N = 6$ per group. (**B**) STRING analysis of gene networks (highest confidence = 0.900; no interactors) among top 200 down-regulated differentially expressed genes (DEGs = adj. *P* value < 0.05) from colonic macrophages in **A**. (**C**) RNAseq gene set enrichment analysis (GSEA) of inflammasome genes in colonic Ly6C^{lo} MHC-II^{+} macrophages in mice treated

as in **c**. *N* = 6 per group. Top 10 leading edge genes shown in heatmap. (**D**) Volcano plot of transmembrane signalling genes (GO:0004888) in colonic macrophages from **A**. (**E**) Bone marrow chimera generation strategy. (**F, G**) Blood reconstitution analysis in 100 % bone marrow chimeric mice at 8 weeks post-irradiation. *N* = 5 per group. Medians indicated. (**H**) Liver CFU in 100 % bone marrow chimeras at 10 dpi. *N* = 5 per group. Mean ± SEM indicated. (**I**) Colonic P2 MNP pro-IL-1β expression in 100 % chimeric mice shown in **F**. Mean ± SEM indicated. (**J**) IL-22^{+} gating and IL-17A quantification of donor colonic CD3ϵ^{-} CD4^{-} ILCs in 100 % chimeric mice at 10 dpi. Mean ± SEM indicated. (**K**) Colonic donor CD19^{+} B cell quantification from mice in **J**. Data representative of two independent experiments. *P* values were calculated using the standard DESeq 2 method with multiple comparisons correction using the BH procedure (**A, D**), Student's t test (**H**), and nonparametric Mann-Whitney U test (**I**). * $P < 0.05$; ** $P < 0.01$; *** $P < 0.001$; **** $P < 0.0001$.

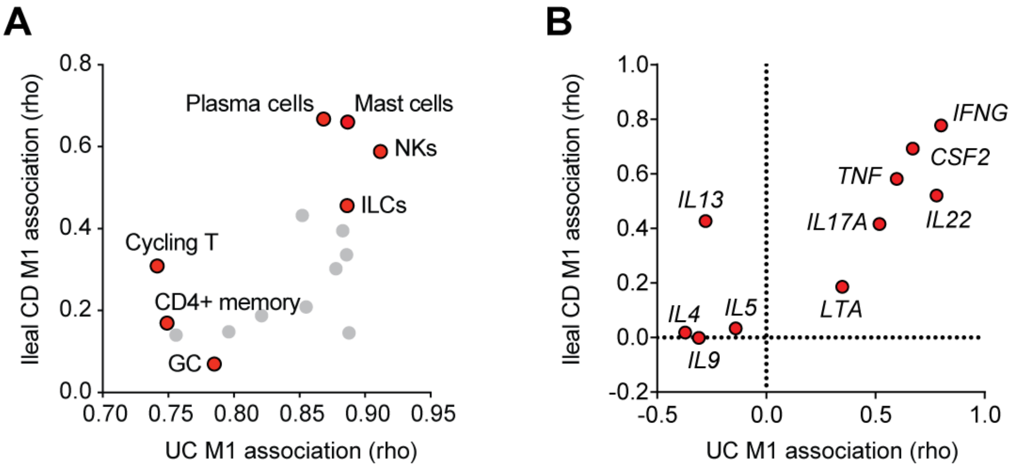

Figure S4. ILCs and GM-CSF regulate intestinal macrophage polarisation. Related to Figure 4. (**A, B**) Immune cell signature (**A**) and cytokine expression (**B**) rho values for correlation with sLPS M1 signature in transcriptomics datasets of UC (GEO: GSE59071) and ileal CD (GEO: GSE93624) mucosal biopsies.

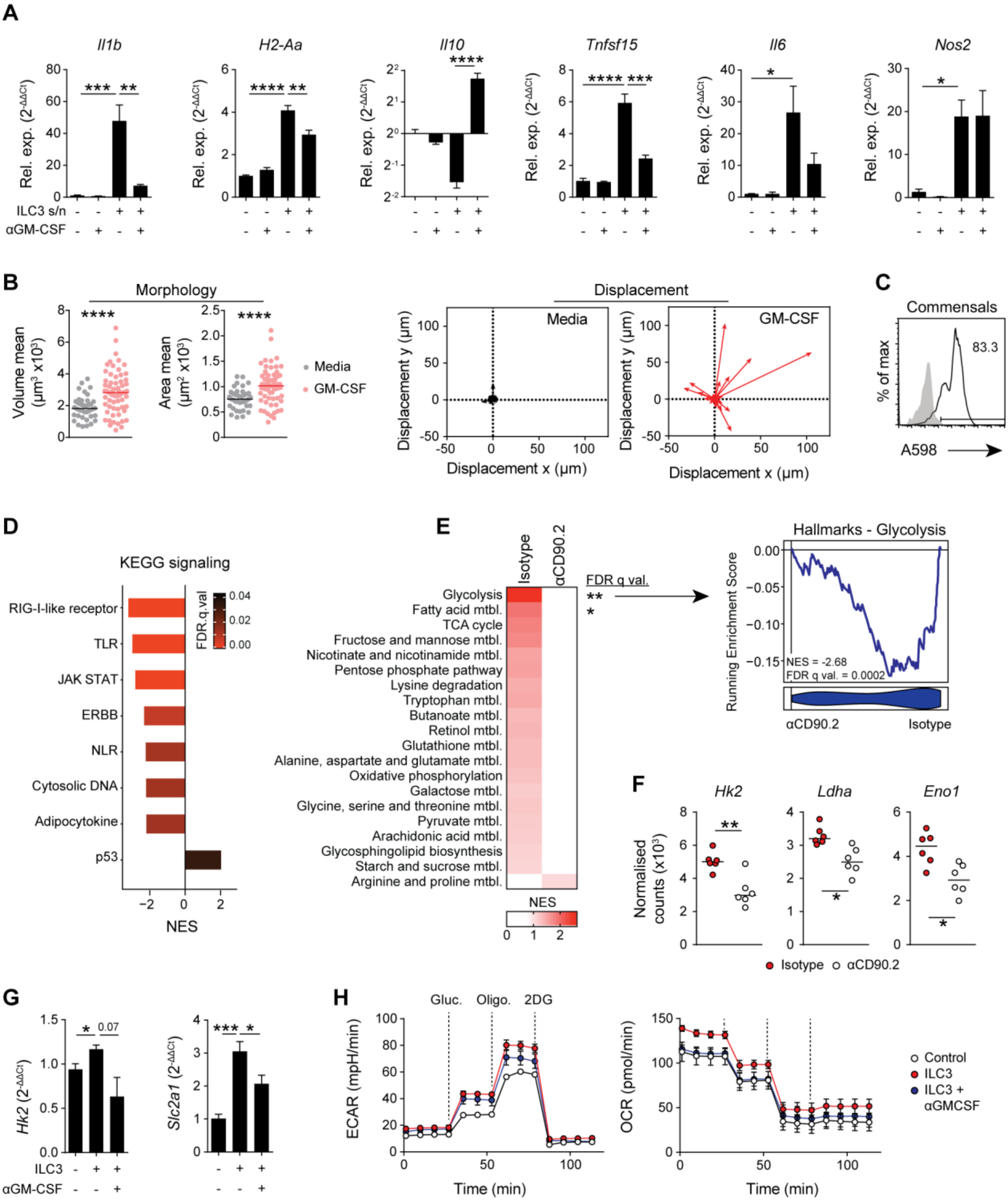

Figure S5. ILC3-derived GM-CSF regulates MNP microbicidal activity and metabolism. Related to Figure 5. (**A**) Cytokine, MHC-II, and NOS2 expression in BMDMs treated with ILC3-conditioned media ± anti-GM-CSF IgG for 16 h. Mean ± SEM indicated. (**B**) Morphology and displacement analysis of control or GM-CSF-primed BMDMs. Medians are indicated. (**C**) Fluorescent labelling of murine intestinal commensals. (**D**) KEGG signalling pathways in colonic macrophages ± anti-CD90.2 IgG administration after 6 days of DSS by RNAseq. (**E**) RNAseq GSEA of Kyoto Encyclopaedia of Genes and Genomes (KEGG) metabolic pathways in colonic Ly6C^{lo} MHC-II^{+} macrophages from *Rag2*$^{-/-}$ mice treated with 2 % DSS ± isotype or anti-CD90.2 IgG for 6 days (*n* = 6 per group). Medians indicated. (**F**) Expression of key glycolysis genes from macrophages in **E**. Medians indicated. (**G**) HK2 and GLUT-1 expression in BMDMs ± ILC3-conditioned supernatant for 16h ± anti-GM-CSF IgG. Mean ± SEM indicated. (**H**) ECAR and OCR trace in BMDMs ± ILC3-conditioned supernatant ± anti-GM-CSF IgG for 16 h. Mean ± SEM indicated. Data are representative of two (**B, H**) or three (**A, G**) independent experiments. *P* values calculated using a one-way ANOVA (**A, G**), Mann-Whitney U test (**B**). * $P < 0.05$; ** $P < 0.01$; *** $P < 0.001$; **** $P < 0.0001$.

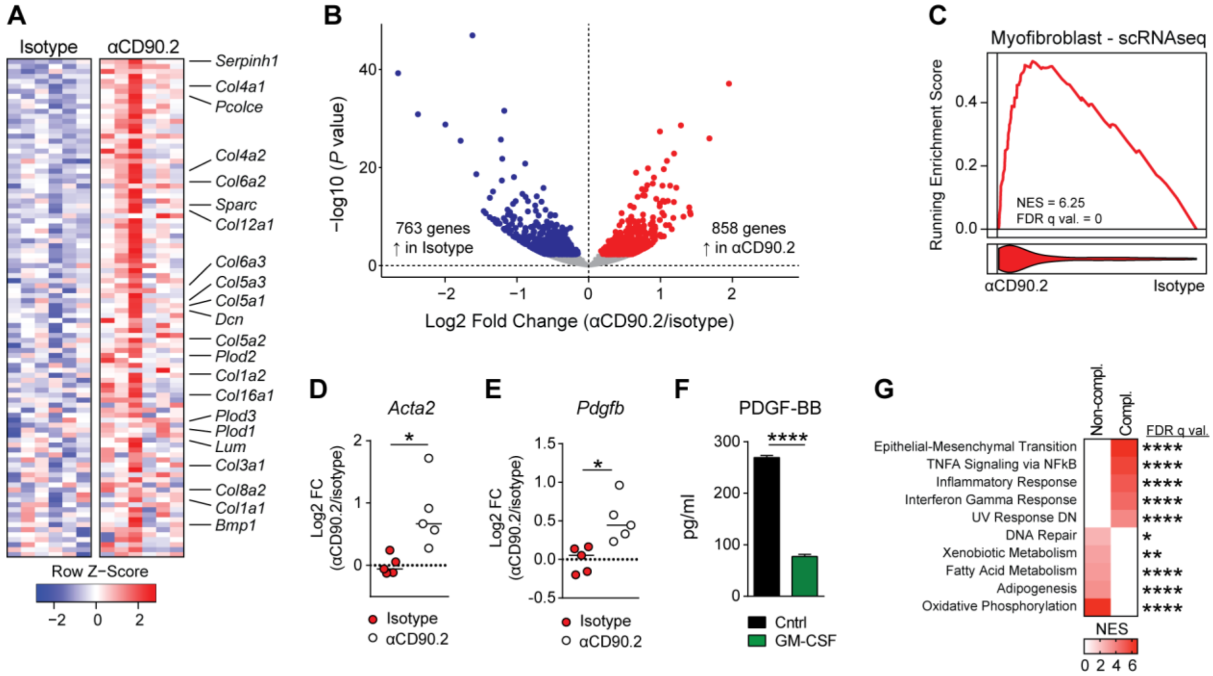

Figure S6. ILCs suppress a pro-repair macrophage phenotype. Related to Figure 6. (**A**) Heatmap of *Epithelial-Mesenchymal Transition* leading edge genes showing enrichment in colonic Ly6C^{lo} MHC-II^{+} macrophages from *Rag2*$^{-/-}$ mice treated with 2 % DSS ± anti-CD90.2 IgG for 6 days (n = 6 per group). Annotated genes are involved in collagen formation or interaction. (**B**) Volcano plot of DEGs in whole colonic tissue from mice treated as in **A**. (**C**) Myofibroblast gene signature GSEA in whole colonic tissue from mice treated as in **A**. (**D**) *Acta2* expression in whole colonic tissue from mice in **A**. Medians are indicated. (**E**) *Pdgfb* expression in whole colon tissue determined by RNAseq in mice treated as in **A**. Medians indicated. (**F**) PDGF-BB ELISA on supernatants from BMDMs treated with control or GM-CSF-supplemented media for 24 h. Mean ± SEM indicated. (**G**) GSEA of Hallmarks pathways in a publicly available transcriptomics dataset of complicated (n = 27) versus non-complicated (n = 183) ileal Crohn's disease biopsies, derived from GEO: GSE93624. Data representative of three independent experiments (**F**). *P* values were calculated by DESeq 2 method with multiple comparisons correction using the BH procedure (**A, B, D, E**), and Student's *t* test (**F**).* $P < 0.05$; ** $P < 0.01$; *** $P < 0.001$; **** $P < 0.0001$.
